# An enhanced jellyfish search optimizer for stochastic energy management of multi-microgrids with wind turbines, biomass and PV generation systems considering uncertainty

**DOI:** 10.1038/s41598-024-65867-8

**Published:** 2024-07-05

**Authors:** Deyaa Ahmed, Mohamed Ebeed, Salah Kamel, Loai Nasrat, Abdelfatah Ali, Mostafa F. Shaaban, Abdelazim G. Hussien

**Affiliations:** 1 Holding Company for Water and Wastewater (HCWW), Aswan, 81542 Egypt; 2 Faculty of Engineering, Sohag University, Sohag, 82524 Egypt; 3 Department of Electrical Engineering, University of Jaén, EPS Linares, 23700 Linares, Jaén Spain; 4 Department of Electrical Engineering, Faculty of Engineering, Aswan University, Aswan, 81542 Egypt; 5 Department of Electrical Engineering, American University of Sharjah, 26666 Sharjah, United Arab Emirates; 6 Department of Electrical Engineering, South Valley University, Qena, 83523 Egypt; 7 Department of Computer and Information Science, Linköping University, Linköping, Sweden; 8 Faculty of Science, Fayoum University, Fayoum, Egypt; 9 MEU Research Unit, Middle East University, 11831, Amman, Jordan

**Keywords:** Energy management, Microgrids, Biomass, Renewable energy, Uncertainty, Mathematics and computing, Information technology, Scientific data, Software

## Abstract

The energy management (EM) solution of the multi-microgrids (MMGs) is a crucial task to provide more flexibility, reliability, and economic benefits. However, the energy management (EM) of the MMGs became a complex and strenuous task with high penetration of renewable energy resources due to the stochastic nature of these resources along with the load fluctuations. In this regard, this paper aims to solve the EM problem of the MMGs with the optimal inclusion of photovoltaic (PV) systems, wind turbines (WTs), and biomass systems. In this regard, this paper proposed an enhanced Jellyfish Search Optimizer (EJSO) for solving the EM of MMGs for the 85-bus MMGS system to minimize the total cost, and the system performance improvement concurrently. The proposed algorithm is based on the Weibull Flight Motion (WFM) and the Fitness Distance Balance (FDB) mechanisms to tackle the stagnation problem of the conventional JSO technique. The performance of the EJSO is tested on standard and CEC 2019 benchmark functions and the obtained results are compared to optimization techniques. As per the obtained results, EJSO is a powerful method for solving the EM compared to other optimization method like Sand Cat Swarm Optimization (SCSO), Dandelion Optimizer (DO), Grey Wolf Optimizer (GWO), Whale Optimization Algorithm (WOA), and the standard Jellyfish Search Optimizer (JSO). The obtained results reveal that the EM solution by the suggested EJSO can reduce the cost by 44.75% while the system voltage profile and stability are enhanced by 40.8% and 10.56%, respectively.

## Introduction

The Micro-grid (MG) can be defined as a small power system that involves different distributed generators (DGs) and energy storage units for providing energy to a certain load like a microturbine, PVs, WTs, small hydro units, diesel, biomass generation systems as well as batteries, super capacitors, Compressed air energy storage, and flywheels^1,2^. The MGs can be classified according to their connection with the main electric system to the on-grid MGs and the off-grid or isolated MGs^3^. In the on-grid MGs, the DGs and the distribution networks are used to provide the energy to the load concurrently while in the isolated type, the load delivers the energy from the DGs only which means that the MGs operate independently^4,5^. Also, the MGs can be classified according to the power system to AC and DC MGs. Furthermore, the AC MGs are divided into two types, single-phase MG and three-phase MGs.

The optimal operation and energy management of the MG has a great attention for a set of reasons including reducing the operation and production energy costs, ensuring the reliable and stable operation state of the MGs, reducing the dependency on fossil fuel-based DGs, and attaining the most sustainable and eco-friendly operation by maximizing utilization of renewable energy (RE) systems. Thus, numerous optimization methods were proposed for the EM solution of the on-grid or off-grid MGs. In^6^, a hierarchical Genetic Algorithm (GA) was presented for the EM solution of a grid-connected MG involving PV/WT/Batteries for profit maximization with demand side response.

The Homer software and the particle swarm optimization were implemented to solve the EM of an isolated MG which consists of PV/diesel/WT/batteries for minimizing total net present cost (TNPC) while considering demand side response (DSR) and different battery technologies^7^. The EM of a DC microgrid consisting of PV and batteries was solved using a modified differential evolution (MDE) to alleviate the operation cost while considering the aging of batteries^8^. The Improved Walrus Optimization Algorithm (I-WaOA) was presented based on Cauchy mutation and the distance fitness balance for the EM of the IEEE 118-bus distribution system (DS) to reduce the all-over costs and system performance enhancement under the DSR and uncertainties of the system^9^. Hachemi et al. solved the EM of the 112‐bus Algerian real DS with the optimal installation of PV and WT-based DGs considering four uncertain parameters using the Modified reptile search algorithm for the cost and the performance system improvement^10^. A modified Capuchin Search Algorithm (MCapSA) was developed based on three modifications for the EM solution of the IEEE 33-bus and 69-bus DSs for multi-objective function including the all-over cost, the voltage deviations (VDs) reduction and under the load-power uncertainty^11^. Khunkitti et al. presented a significant study using the Marine Predators Algorithm (MaMPA) as an effective algorithm to solve the EM problem for many objective functions including the total cost, transmission losses, emissions, and the voltage stability index of the IEEE 30- and 118-bus systems^12^. The Prairie Dog Optimization Algorithm (PDO) has been formulated by AE Ezugwu el al. as an efficient algorithm where its experimental results proved its efficiency when compared with other algorithms with a good balance of exploitation and exploration in solving the EM problem^13^. In^14^, the authors presented a comprehensive review of applications for the Artificial Hummingbird Algorithm (AHA) for different optimization problems like energy management, image processing, optimal control, and engineering design applications. Krill herd optimization and the ant lion optimizer were employed to solve the EM of an MG consisting of PV/batteries /fuel cell (FC)/WT to reduce the cost^15^. The artificial hummingbird algorithm was utilized for the inclusion of PVs and WTs on the IEEE 33-bus network for reducing emissions, costs, VDs, and VS enhancement^16^. Shadman Abid et al. has been utilized for EM of DSs including IEEE 33-bus and 69-bus for multi-objective functions losses, voltage stability margin, economic savings, and VDs^17^. The EM of EEE 33-bus and 69-bus was solved with the integration of PVs, virtual synchronous generators, and WTs for alleviating the frequency deviation and maximizing energy saving using multi-objective PSO (MOPSO)^18^. The Equilibrium Optimizer was suggested for solving the EM for a 12-bus small DS for a multi-objective function involving the total cost, VD, and VS in the presence of the WTs and PVs under PV/WT/Load powers uncertainty^19^.

Recently, the new policy of the electric sectors has been to split the large-scale grids into small multi-micro grids to maximize the use of the DGs to ensure flexible operation and provide a simple and decentralized control ability^20,21^. The EM was solved for MMGs using a two-level framework based on reinforcement learning taking into consideration the time-varying of the generated power by PV panels and the load demand^22^.

Recently, the electric sectors have been prone to split large-scale grids into small multi-micro grids to maximize the use of the DGs to ensure flexible operation and provide a simple and decentralized control ability^20,21^. The EM of an MMG was solved using an improved sparrow search algorithm for cost and emissions reductions^23^. In^24^, a 33-bus DS was divided into three MMGs, and the EM was solved to diminish the total cost with DSR using the game theory. The EM of isolated MMG was solved using mixed integer linear programming with DSR and the uncertainties of the system were presented using the scenario-based method^25^. The authors in^26^ solved the EM of an MMG as a stochastic bi-level problem with the integration of multiple energy sources for reducing the total costs considering the uncertainties of the system.

Recently, the utilization of biomass energy sources has wildly increased as a renewable and sustainable energy source^27^. The authors in^28^ discussed the evolution of using biomass as a renewable energy resource during the eighteenth and nineteenth centuries. Thus, the biomass-based DGs are wildly allocated in the distribution and the MGs along with other sources^29–35^. Table 1 summarizes the recent papers on the EM solution.

**Table 1:** Methods of the EM solution.

Reference	Optimizer	DGs			Uncertainty	Description	Functions	Year
		WT	PV	Biomass				
^6^	GA	✓	✓	✗	✗	Centralized	Cost	2020
^7^	Homer/PSO	✓	✓	✗	✗	Centralized	Cost	2020
^8^	MDE	✗	✓	✗	✗	Centralized	Cost	2015
^9^	I-WaOA	✓	✓	✗	✓	Centralized	Cost/VD/VSI	2023
^10^	MRSA	✓	✓	✗	✓	Centralized	Cost/VD/VSI	2023
^11^	MCapSA	✓	✓	✗	✓	Decentralized	Cost/VD/VSI	2023
^15^	KH, ALO	✓	✓	✗	✗	Centralized	Cost/emission	2018
^16^	AHA	✓	✓	✗	✓	Centralized	Cost/VD/VSI/emission	2023
^17^	AHA	✓	✓	✗	✓	Centralized	PL/VD/VSI	2022
^18^	MOPSO	✓	✓	✗	✗	Centralized	FD/Cost	2023
^19^	EO	✓	✓	✗	✓	Centralized	Cost/VD/VSI	2021
^20^	MMGEMS	✓	✓	✗	✓	Decentralized	FD/VD/VSI	2021
^22^	DSO, OPF	✓	✓	✗	✓	Decentralized	Cost /PL	2022
^23^	ISSA	✓	✓	✗	✗	Decentralized	Cost/emission	2022
^24^	Game theory	✓	✓	✗	✓	Decentralized	Cost	2022
^25^	MILP	✓	✓	✗	✓	Decentralized	Cost	2020
^26^	DNO	✓	✓	✗	✓	Decentralized	Cost	2014
^27^	SOA, SMA	✓	✓	✓	✗	Centralized	Cost/LPSP	2021
^29^	MOPSO	✗	✗	✓	✗	Centralized	Cost/LPSP	2021
^31^	MILP	✓	✓	✓	✗	Centralized	Cost	2011
^32^	RT_Lab	✗	✓	✓	✓	Centralized	Cost	2016
^33^	HOMER	✓	✓	✓	✗	Centralized	Cost	2015
^34^	ACO	✓	✓	✓	✗	Centralized	Cost/emission	2018
^35^	HOMER	✓	✓	✓	✗	Centralized	dumped energy	2009
Our work	EJSO	✓	✓	✓	✓	Decentralized	Cost/VD/VSI	

The JSO optimizer was utilized for addressing several optimization techniques. However, it suffers from stagnation in the case of solving the high non-convex equations. An enhanced version of JSO was proposed for tackling this issue. The first modification is the FDB^36^ which was implemented to different optimization techniques like the artificial rabbits optimization algorithm^37^, the Lévy flight distribution algorithm^38^, An adaptive gaining-sharing knowledge algorithm^39^, the Artificial Hummingbird Algorithm^40^, reptile search algorithm ^10^. Additionally, the second modification strategy is based on Weibull Flight Motion (WFM) which was implemented to improve the exploration strategy of optimization methods^41^.

The vital importance of this work is that solving the energy management of multi-microgrids with optimal integration of renewable energy sources can decrease the dependence of using conventional sources as well as enhance the performance of the system.

This paper can fill the research gap where EJSO is implemented for EM of MMGs with the integration of PV, biomass, and WTs simultaneously under uncertainties of the loading and the output powers of the renewable energy resources (RERs) for both economic and technical objective functions. However, the novelties and the contributions can be depicted as follows:

- The energy management of a multi of multi-microgrids of the 85-bus system is solved with optimal integration of a hybrid system including PV, WT, and biomass units.
- The uncertainties of the system such as wind speed, load power, and solar irradiance are considered in the energy management solution.
- Proposing A novel enhanced Jellyfish Search Optimizer (EJSO) for solving the EM based on (WFM) and (FDB) to tackle the stagnation problem of the conventional JSO technique.

The searching ability of the suggested EJSO is demonstrated using CEC -2019 and the traditional benchmark functions. In addition,a comprehensive comparison with SCSO, DO, GWO, the standard JSO, and WOA are achieved.

The remaining sections are listed as follows: "Problem formulation" section gives a deep clarification about the objective functions and the related constraints. "Uncertainty modeling" section describes the method of representing the uncertainties in the system. "Jellyfish search optimizer (JSO)" and "The enhanced jellyfish search optimizer (EJSO)" sections give the description and mathematical equations of JSO and the EJSO, respectively. The discussion of the yielded results is depicted in "Simulation results" section while the summarization of the conclusions is outlined in the final section.

## Problem formulation

### Objective functions

#### The cost reduction

The first function considered is the total cost ($TC$), which involves the cost of the energy supplied from the main grid (${C}_{1}$), the energy loss cost (${C}_{2}$), the PV cost (${C}_{3}$), the WT unit cost (${C}_{4}$), the biomass cost (${C}_{5}$) and, the $TC$ can be expressed as follows:

1 $$TC={C}_{1}{+{C}_{2}+C}_{3}+{C}_{4}+{C}_{5},$$

In which

2 $${C}_{1}= 365\times {U}_{Gr}\times \sum_{h=1}^{24}{P}_{Gr}(h),$$

where ${P}_{Gr}$, and ${U}_{Gr}$ are the delivered power from the grid and the cost of this power per kW, respectively.

3 $${C}_{2}=365\times {U}_{L}\times \sum_{h=1}^{24}{P}_{T\_L}(h),$$

where ${P}_{T\_L}$ and ${U}_{L}$ are the power loss and its cost per kW, respectively.

4 $${C}_{3}= {C}_{PV}^{inv.}+{C}_{PV}^{O\&M},$$

where ${C}_{PV}^{inv.}$ denotes the investment cost of the PV panels, ${C}_{PV}^{O\&M}$ is its operation and maintenance cost.

5 $${C}_{PV}^{O\&M}={U}_{PV}^{O\&M}\times \sum_{h=1}^{24}{P}_{PV}(h),$$

6 $${C}_{PV}^{inv.}={CF\times {U}_{PV}\times P}_{r\_PV}$$

where $${U}_{PV}^{O\&M}$$, $${U}_{PV}$$, $${P}_{PV}$$, $${P}_{r\_PV}$$ refer to operation and maintenance cost, i.e., $/kWh, the investment cost, i.e., $/kW, the output and rated powers of the PV panels, respectively. $$CF$$ is the capital recovery factor. The WT’s cost can be expressed using (7).

7 $${C}_{4}= {C}_{WT}^{inv.}+{C}_{WT}^{O\&M},$$

where ${C}_{WT}^{inv.}$ denotes the investment cost of the PV panels, ${C}_{PV}^{O\&M}$ is its operation and maintenance cost.

8 $${C}_{WT}^{O\&M}={U}_{WT}^{O\&M}\times \sum_{h=1}^{24}{P}_{WT}(h),$$

9 $${C}_{W}^{inst.}={CF\times {E}_{WT}\times P}_{r\_WT},$$

where $${U}_{WT}^{O\&M}$$, $${U}_{WT}$$, $${P}_{WT}$$, $${P}_{r\_WT}$$ refer to the operation and maintenance cost of the WT in $/kWh, the investment cost in $/kW, and the output and rated powers of the PV panels, respectively.

The cost of the biomass system can be calculated as follows:

10 $${C}_{PV}^{O\&M}={U}_{PV}^{O\&M}\times \sum_{h=1}^{24}{P}_{PV}(h),$$

11 $${C}_{PV}^{inv.}={CF\times {U}_{PV}\times P}_{r\_PV}$$

where $${U}_{PV}^{O\&M}$$, $${U}_{PV}$$, $${P}_{PV}$$, $${P}_{r\_PV}$$ refer to operation and maintenance cost, i.e., $/kWh, the investment cost, i.e., $/kW, the output and rated powers of the PV panels, respectively. $$CF$$ is the capital recovery factor. The WT’s cost can be expressed using (7).

12 $${C}_{4}= {C}_{WT}^{inv.}+{C}_{WT}^{O\&M},$$

where ${C}_{WT}^{inv.}$ denotes the investment cost of the PV panels, ${C}_{PV}^{O\&M}$ is its operation and maintenance cost.

13 $${C}_{WT}^{O\&M}={U}_{WT}^{O\&M}\times \sum_{h=1}^{24}{P}_{WT}(h),$$

14 $${C}_{W}^{inst.}={CF\times {U}_{WT}\times P}_{r\_WT},$$

where $${U}_{WT}^{O\&M}$$, $${U}_{WT}$$, $${P}_{WT}$$, $${P}_{r\_WT}$$ refer to the operation and maintenance cost of the WT in $/kWh, the investment cost in $/kW, and the output and rated powers of the WT, respectively.

The cost of the biomass system cost can be expressed using (15).

15 $${C}_{4}= {C}_{bio}^{inv.}+{C}_{bio}^{O\&M},$$

where ${C}_{bio}^{inv.}$ denotes the investment cost of the PV panels, ${C}_{bio}^{O\&M}$ is its operation and maintenance cost.

16 $${C}_{bio}^{O\&M}={U}_{bio}^{O\&M}\times \sum_{h=1}^{24}{P}_{bio}(h),$$

17 $${C}_{W}^{inst.}={CF\times {U}_{bio}\times P}_{r\_bio},$$

where $${U}_{bio}^{O\&M}$$, $${U}_{bio}$$, $${P}_{bio}$$, $${P}_{r\_bio}$$ refer to the operation and maintenance cost of the biomass system in $$\$/\text{kWh}$$, the investment cost in $/kW, and the output and rated powers of the biomass system, respectively. The recovery factor can be obtained using (18).

18 $$CF=\frac{{\beta }_{PV,WT,bio}\times {(1+{\beta }_{PV,WT,bio})}^{{NP}_{PV,WT,bio}}}{{(1+{\beta }_{PV,WT,bio})}^{{NP}_{PV,WT,bio}}-1},$$

$NP$ is the lifetime of the generation unit. $\beta$ is the interest rate.

The yielded power from the PV panels is formulated using (19)^42^:

19 $${P}_{PV}\left(ir\right) =\left\{\begin{array}{ll}{P}_{r\_ PV} \left(\frac{{ir}^{2}}{{{ir}^{2}}_{std }\times {X}_{s} }\right) & \quad for \; 0<{ir}^{2}\le {X}_{sp}\\ { P}_{r\_ PV}\left(\frac{ir}{{ir}_{std}}\right) & \quad for \; {X}_{s}\le {ir}^{2}\le {ir}_{std}\\ { P}_{r\_ PV} & \quad {ir}_{std} \le {ir}^{2} \end{array}\right.$$

where ${ir}_{std}$ and $ir$ denote solar irradiance and the standard deviations which equals 1000 W/m^2^.

The WT's output power can be computed as follows^43^:

20 $${P}_{W}\left(W\right)=\left\{\begin{array}{lll}0& for& W<{W}_{cin} \; and \; W > {W}_{o}\\ {P}_{r\_w}\left(\frac{W-{W}_{cin}}{{W}_{rs}-{W}_{cin}}\right)& for&\left({W}_{cin}\le W\le {W}_{rs}\right)\\ {P}_{rated\_wind}&for & \left({W}_{rs} < W\le {W}_{cout}\right)\end{array}\right.$$

where, ${W}_{cout}$, ${W}_{cin}$ and ${W}_{rs}$ represent the cut-out, cut-in, and rated wind speed respectively.

#### Voltage profile enhancement

Minimizing the voltage deviations will improve the performance of the system. The voltage deviation can be expressed as follows:

21 $$\sum VD=\sum_{k=1}^{24}\sum_{n=1}^{Nb}\left|\left({V}_{n}-1\right)\right|$$

where $Nb$ refers to the number of buses in the MMGs.

#### Voltage stabilization improvement

Maximization of the voltage stability index (VSI) can improve the system performance^43^:

22 $${VSI}_{n}={\left|{V}_{n}\right|}^{4}-4{\left({P}_{n}{X}_{n}-{Q}_{n}{R}_{n}\right)}^{2}-4\left({P}_{n}{X}_{n}+{Q}_{n}{R}_{n}\right){\left|{V}_{n}\right|}^{2},$$

23 $$\sum VSI=\sum_{h=1}^{24}\sum_{n=1}^{NB}{VSI}_{n},$$

where ${X}_{n}$ and ${R}_{n}$ represent the reactance and the resistance of the *n*-th line, respectively. ${P}_{n}$ and ${Q}_{n}$ are the injected real and reactive powers, respectively.

The previous objective functions can be taken into consideration concurrently as depicted in (24).

24 $${f}_{t}= {a}_{1}\times {fun}_{1}+ {a}_{2}\times {fun}_{2}+{a}_{3}\times {fun}_{3},$$

25 $${fun}_{1}=\frac{{Co}_{Rs}}{{Co}_{Base}},$$

26 $${fun}_{2}=\frac{{\sum VD}_{Rs}}{{\sum VD}_{Base}},$$

27 $${fun}_{3}=\frac{1}{{\sum VSI}_{Rs}}.$$

$Rs$ and $Base$ are subscripts that refer to the system with and without PVs and WTs, respectively. ${a}_{1},$ ${a}_{2}$ and ${a}_{3}$ are parameters that were selected to be 0.5, 0.25, and 0.25, respectively^44^.

### The constraints

#### Inequality constraints

28 $${V}_{Min}\le {V}_{i}\le {V}_{Max},$$

29 $${P}_{PV\_r}+{P}_{win{d}_{\_r} }{+P}_{bio\_r}\le \sum_{i=1}^{NB}{P}_{Load,i},$$

30 $${PF}_{Min\_w}\le PF\le {PF}_{Max\_w},$$

31 $${PF}_{Min\_bio}\le PF\le {PF}_{Max\_bio},$$

32 $${I}_{n}\le {I}_{max,n} n =\text{ 1,2},3\dots ,NT,$$

where the upper and the lower limits for the voltage are ${V}_{min}$, and ${V}_{max},$ respectively, while. ${P}_{Load}$ and ${\text{Q}}_{\text{Load}}$ denote the real and reactive power for the load respectively. ${I}_{max,n}\text{ is}$ the maximum limit of the current at the *n*-th line. ${PF}_{Min\_w}$ and ${PF}_{Max\_w}$ refer to the minimum and the maximum boundaries for the power factors for the WT, while ${PF}_{Min\_bio}$ and ${PF}_{Max\_bio}$ are the upper and lower limitations for the biomass power factor. $NT$ is the number of the TLs.

#### Equality constraints

33 $${P}_{S}+{P}_{PV}+{P}_{Wind}+ {P}_{bio} = \sum_{i=1}^{NT}{P}_{Losses,i}+\sum_{i=1}^{NB}{P}_{Load,i},$$

34 $${Q}_{S}+{Q}_{Wind}+{Q}_{bio} = \sum_{i=1}^{NT}{Q}_{Losses,i}+\sum_{i=1}^{NB}{Q}_{Load,i},$$

where, ${P}_{S},{P}_{PV},{P}_{Wind}\text{ and }{P}_{bio}$ are the purchased powers of the utility network, the PV units, the units WTs, and the biomass, respectively. ${Q}_{S}, {Q}_{Wind}\text{ and }{Q}_{bio}$ are the reactive powers for the main substation, the WTs, and the biomass units respectively.

## Uncertainty modeling

In this work, energy management is solved by taking into consideration three stochastic parameters. The probability density functions (PDFs) are utilized for the representation uncertainties of these parameters.

The first stochastic parameter is the loading which is varied at each time discrete *t* and it is represented in terms of the Normal PDF based on the standard deviation (${\sigma }_{PL}$) of the load and its average value (${\mu }_{PL}$) as follows^45,46^:

35 $$f\left({P}_{L}\right)=\frac{1}{\sqrt{2\pi {\sigma }_{PL}}}\text{exp}\left(-\frac{{\left({P}_{L}-{\mu }_{PL}\right)}^{2}}{{2}^{\times }{\sigma }_{PL}^{2}}\right).$$

The wind speeds in any area vary randomly. Weibull distribution is utilized for describing the random variation of the wind speed. Weibull distribution pdf at time discrimination *t* is described using two parameters including the shape (${k}_{t}$) and the scale (${c}_{t}$) factors that are driven by the standard deviation (${\sigma }_{t}^{v}$) and average (${\mu }_{t}^{v}$) of the wind speed as follows^47,48^:

36 $${f}_{t}^{\text{WT}}(v)=\frac{{k}_{t}}{{c}_{t}}{\left(\frac{{v}_{t}}{{c}_{t}}\right)}^{{k}_{t}-1}\text{exp}\left(-{\left(\frac{{v}_{t}}{{c}_{t}}\right)}^{{k}_{t}}\right),$$

in which

37 $${k}_{t}={\left(\frac{{\sigma }_{t}^{v}}{{\mu }_{t}^{v}}\right)}^{-1.086},$$

38 $${C}_{t}=\frac{{\mu }_{t}^{v}}{\Gamma \left(1+\left(1/{k}_{t}\right)\right)},$$

The third uncertain parameter is the solar irradiance which is modeled using Beta PDF as depicted in (39). The Beta PDF is described using two parameters including the shape factors (*α, β*) which can be assigned using the average and the standard deviation of the irradiance (${\mu }_{S}^{t}$, ${\sigma }_{S}^{t}$)^49^:

39 $${f}_{s}^{t}(s)=\frac{\Gamma \left({\alpha }^{t}+{\beta }^{t}\right)}{\Gamma \left({\alpha }^{t}\right)\cdot\Gamma \left({\beta }^{t}\right)}\cdot {\left({s}^{t}\right)}^{{\alpha }^{t}-1}\cdot {\left(1-{s}^{t}\right)}^{{\beta }^{t}-1}\text{for }{\alpha }^{t}>0; {\beta }^{t}>0,$$

40 $${\beta }^{t}=\left(1-{\mu }_{S}^{t}\right)\times \left(\frac{{\mu }_{S}^{t}\left(1+{\mu }_{S}^{t}\right)}{{\left({\sigma }_{S}^{t}\right)}^{2}}-1\right),$$

41 $${\alpha }^{t}=\frac{{\mu }_{S}^{t}\times {\beta }^{t}}{\left(1-{\mu }_{S}^{t}\right)} .$$

**Figure 1:**
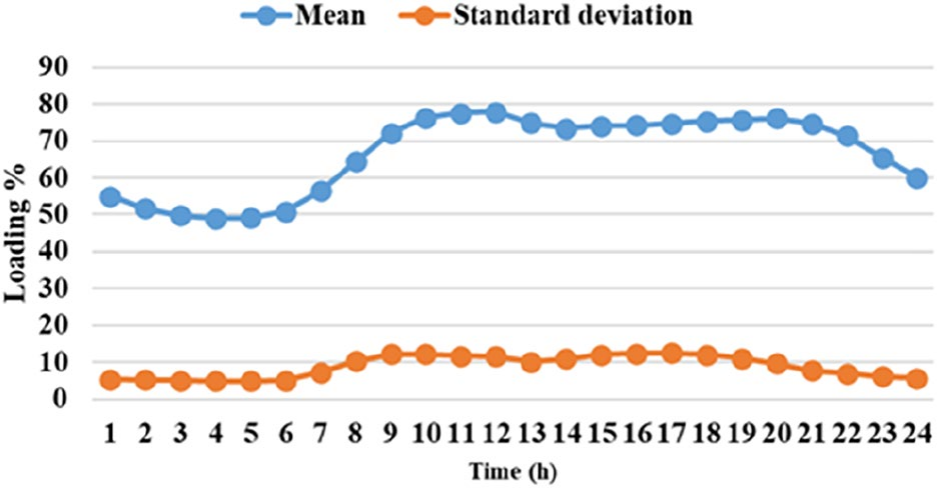
The average load demands and the corresponding standard deviation**.**

**Figure 2:**
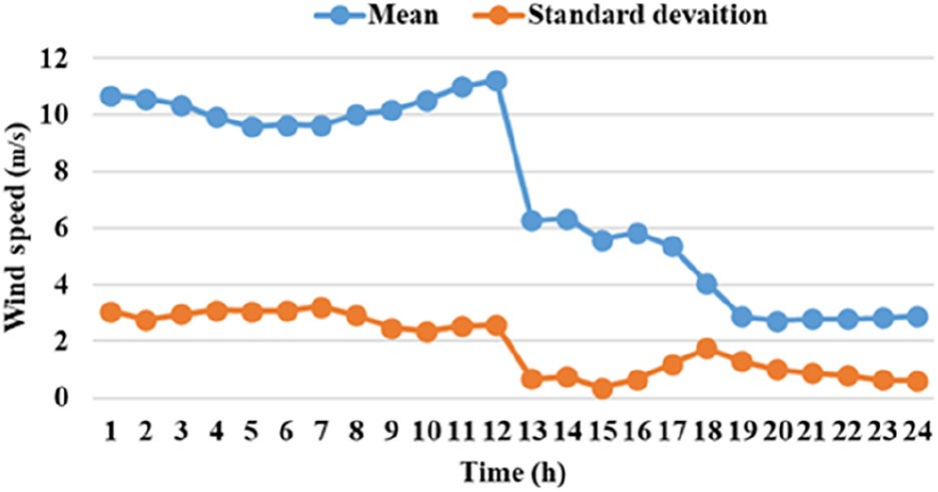
The average wind speed and the corresponding standard deviation.

**Figure 3:**
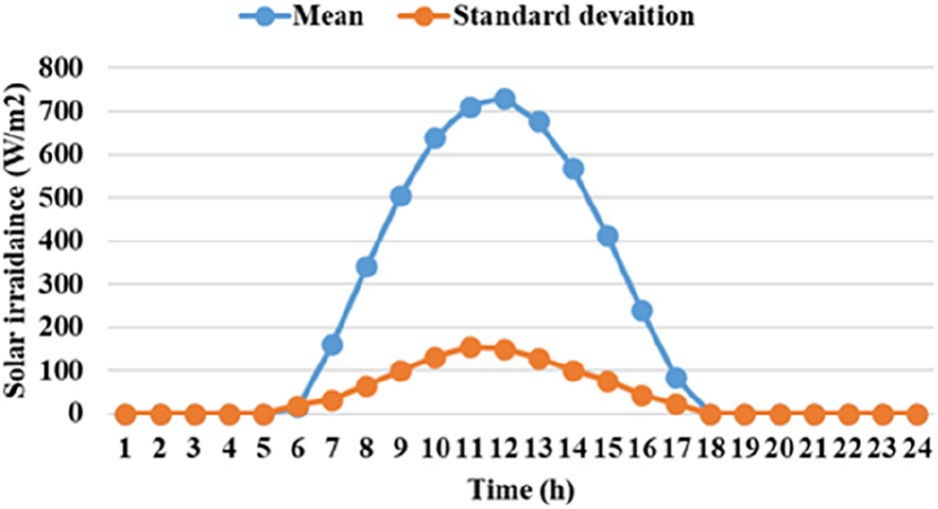
The mean solar irradiance and the corresponding standard deviation.

The means and the standard deviations of the demand, the irradiance, and the wind speed during the day ahead are presented in Figs. 1, 2, and 3, respectively.

**Figure 4:**
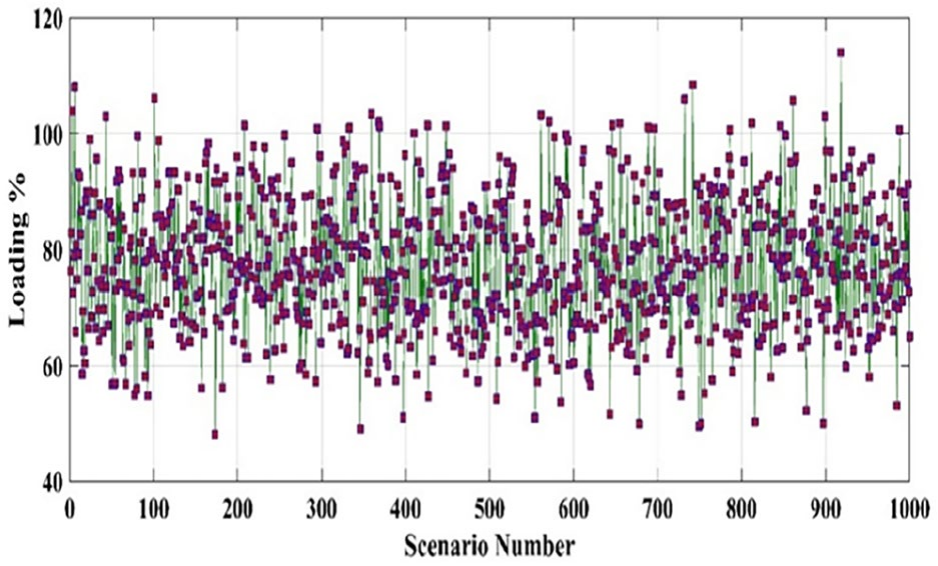
The 1000 generated scenarios by MCS of the load demand at 12:00 PM.

**Figure 5:**
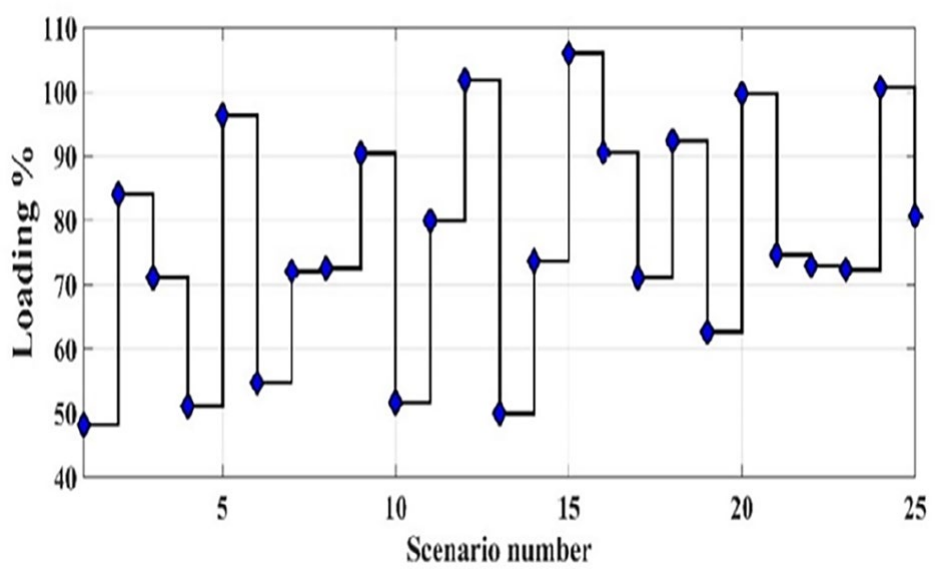
The reduced scenarios by the SBR method of the load demand at 12:00 PM.

For each time segment *t*, The Monte Carlo simulation (MCS) method is employed to obtain 1000 scenarios of the uncertain parameters^50^. Then the scenario-based reduction (SBR) method is employed to minimize the generated scenarios to 25 scenarios^51^. Figure 4 shows the 1000 created scenarios by MCS, while Fig. 5 shows the reduced scenarios by the SBR method.

## Jellyfish search optimizer (JSO)

Jellyfish live all over the world in the water, at different depths and different degrees of temperature. Where jellyfish have different types, but these types differ in size and shape. The behavior of obtaining food differs between jellyfish, as some types of jellyfish obtain food by hunting prey, and other types bring their food by using their tentacles, while the last types obtain their food through filter feeding as they feed on any food carried by the current. It is noted that Jellyfish are known to be weak swimmer organisms, while their orientation concerning currents is the way to their prosperity. Many factors govern the formation of jellyfish swarms, which are those factors related to available nutrients, oxygen availability, and temperatures. The most important factor of these factors is the ocean currents, which work to collect the swarms of jellyfish. In addition to the fact that jellyfish change their location in a swarm or follow ocean currents, jellyfish use a specific mechanism to set the time. The regulation of switching among these types of movement depends on this mechanism. Here, an optimizer was enhanced based on the attitude of jellyfish in searching for food. The jellyfish optimization technique simulates the active and passive motion of the swarm of the jellyfish and its transition between these movements. The suggested algorithm depends on three ideal principles as follows^52^:

1. Jellyfish move with the current of an ocean or move within a swarm.
2. Jellyfish move in searching for food in the ocean, where jellyfish are attracted to those sites that have an abundance of food.
3. The food’s quantity is explained according to the place and corresponding to the objective function.

### Ocean current

Jellyfish are carried away by ocean currents, where the direction of ocean currents is expressed as (DR). This is done by calculating the average vector between jellyfish location within the ocean and the best location of jellyfish, and this can be illustrated as follows:

42 $$\begin{aligned} \overline{DR } & =\frac{1}{{n}_{\text{Pop }}}\sum_{i=1}^{{n}_{\text{P }}}{\text{DR}}_{i}=\frac{1}{{n}_{\text{P }}}\sum_{i=1}^{{n}_{\text{Pop }}}\left({X}_{b}-{e}_{c}{X}_{i}\right)\\ & ={X}_{b}-{e}_{c}\frac{\sum_{i=1}^{{n}_{\text{P }}}{X}_{i} }{{n}_{\text{P }}}={X}_{b}-{e}_{c}\mu \\ \end{aligned}$$

43 $$\text{Set }df={e}_{c}\mu$$

44 $$\overline{DR }={X}_{b}-df$$

where, ${X}_{b}$ represents the optimal position of jellyfish in the swarm, ${n}_{\text{Pop}}$ is the jellyfish number, ${e}_{c}$ denotes the governing attraction factor, $\upmu$ denotes the mean location for all jellyfish and $\text{df}$ represents the difference between the mean place and the current optimal place of the jellyfish. Due to the common distribution of all positions, the jellyfish distance $\pm \beta \sigma$ is the distance around the average site which contains a given certain probability of all jellyfish. Hence, $\text{df}$ can be expressed as:

45 $$df=\beta \times \mu \times \varepsilon$$

By substitution of $df$ from (41) into (40). $\varepsilon$ denotes a variable that is generated randomly from where ${\varvec{\upvarepsilon}}$ ~ Bernoulli(p), with p = 1/2.

46 $$\overline{DR }={X}_{b}-\beta \times \mu \times \varepsilon$$

Based on the foregoing, the new position of the jellyfish can be illustrated as follows:

47 $${X}_{i}(t+1)={X}_{i}(t)+\varepsilon \times \overline{DR }$$

By substituting of $\overline{DR }$ from (45) in (47).

48 $${X}_{i}(t+1)={X}_{i}(t)+\varepsilon \times \left({X}_{b}-\beta \times \mu \times rand\right)$$

### The Jellyfish swarm

Two kinds of motions govern the movement of jellyfish in the group. The two kinds of these movements can be described as follows:

Motion A is the passive movement, and the second type B is the active movement. Jellyfish begin to transfer according to the first type (A), and over time, Jellyfish follow the second type of movement (B). The jellyfish move its position can be presented as follows:

49 $${X}_{i}^{t+1}={X}_{i}^{t}+\gamma \times \varepsilon \times \left({U}_{up}-{L}_{low}\right)$$

where ${U}_{up}$ and ${L}_{low}$ denote the upper boundary and lower boundary of the variables, respectively. $\gamma >0$ refers to the movement factor.

To characterize B movement, two jellyfish (*j, i*) have been chosen where j ≠ i. Where the movement of jellyfish can be described according to the availability of the amount of food. When the available food is high for *j-*th jellyfish more than that for *i-*th jellyfish, this jellyfish moves towards *j-*th jellyfish. The opposite happens if the amount of food is little, the jellyfish (i) shifts away from the *j-*th jellyfish. Based on the above, this movement can be described as follows:

50 $$\overline{ST }={X}_{i}^{t+1}- {X}_{i}$$

where,$\overline{ST }$ denotes the distance and can be expressed as follows:

51 $$\overline{ST }=rand\times \overline {{\text{Direction}}}$$

52 $${X}_{i}^{t+1}={X}_{i}^{t}+\overline{ST }$$

53 $$\overline {{\text{Ei}}}=\left\{\begin{array}{ll}{X}_{j}(t)-{X}_{i}(t) & \quad if \; f\left({X}_{i}\right)\ge f\left({X}_{j}\right)\\ {X}_{i}(t)-{X}_{j}(t)& \quad if \; f\left({X}_{i}\right)<f\left({X}_{j}\right)\end{array}\right.$$

where, $\overline {{\text{Ei}}}$ is the direction of jellyfish motion.

Note that the mechanism of the controlled time is used to explain the kind of movement all over time. As it is not only controlling the motions of A and B within the swarm, but also controlling the motions of jellyfish towards the ocean currents, and this is what will be presented in the next section.

### Mechanism of time control

The transition motion of jellyfish between all types of motions (type A, type B, ocean current) can be explained by the function of time control as expressed in (54).

54 $$cont(t)=\left|\left(1-\frac{t}{{it}_{max}}\right)\times (2\times \varepsilon -1)\right|$$

where $cont$ represents the time control function and it fluctuates from 0 to 1 which compared with a constant value ${C}_{0}$ where, ${C}_{0}=0.5$. ${it}_{max}$ refers to the maximum number of iterations. In case the value of the $count(t)$ is more than ${C}_{0}$, jellyfish move with the ocean current. It is worth noting here that, the jellyfish populations are randomly generated based on a stochastic and logistic map to enhance the initial population diversity and this can be explained as follows:

55 $${\xi }^{{{\prime}}}=\mu \zeta (1-\xi )$$

56 $${X}_{i}(t+1)={X}_{i}(t)+{\xi }^{{{\prime}}}\times \left({U}_{b}-{L}_{b}\right)$$

where $\xi$ denotes a stochastic value that is generated at the start of the iteration in the range [0–1]. $\mu$ has a fixed value where its value equals 4 and ${\xi }^{{{\prime}}}$ is a stochastic and a logistic value where ${\xi }^{{{\prime}}}\ne \left\{0.0, 0.25, 0.75, 0.5, 1.0\right\}$.

## The enhanced jellyfish search optimizer (EJSO)

The EJSO depends upon two improvement approaches including the WFM and the FDB to boost the searching capability of the presented algorithm. The WFM is conceptualized from Weibull Distribution which is based on the scale and the shape factors as explained before^53^. The motion of the jellyfish based on the Weibull flight can be described as follows:

57 $${{\varvec{x}}}_{\text{new,i }}={{\varvec{x}}}_{\text{new,i }}+\text{St}$$

in which

58 $$\text{St= rb (1,1,[1,}D])\cdot \times \text{sign}(\text{rand}(1,D)-0.5),$$

where ${\varvec{r}}{\varvec{b}}\text{ refers}$ to a variable that is randomly obtained from the Weibull distribution. Sign () generates a vector of –1 and + 1. The second strategy is the FDB selection method which was applied to improve several^36,54–57^. The FDB is utilized for enriching the population’s diversity. The FDB is determined by the distance between the best population and the candidates’ solution. Initially, the candidate populations and the corresponding objective functions vector are represented using Eqs. (59) and (60).

59 $$P\equiv {\left[\begin{array}{ccc}{x}_{11}& \cdots & {x}_{1n}\\ \vdots & \ddots & \vdots \\ {x}_{m1}& \cdots & {x}_{mn}\end{array}\right]}_{mxn},$$

60 $$F\equiv {\left[\begin{array}{c}{f}_{1}\\ \vdots \\ {f}_{m}\end{array}\right]}_{mx1},$$

The distance between the best population and the candidate populations is calculated as follows:

61 $${D}_{{P}_{i}}=\sqrt{{\left({x}_{1[i]}-{x}_{1[\text{ best }]}\right)}^{2}+{\left({x}_{2]i]}-{x}_{2[\text{best }]}\right)}^{2}+\cdots +{\left({x}_{n[i]}-{x}_{n[\text{best }]}\right)}^{2}},$$

The fitness distance vector

62 $$DP\equiv {\left[\begin{array}{c}{d}_{1}\\ \vdots \\ {d}_{m}\end{array}\right]}_{mx1}$$

**Figure 6:**
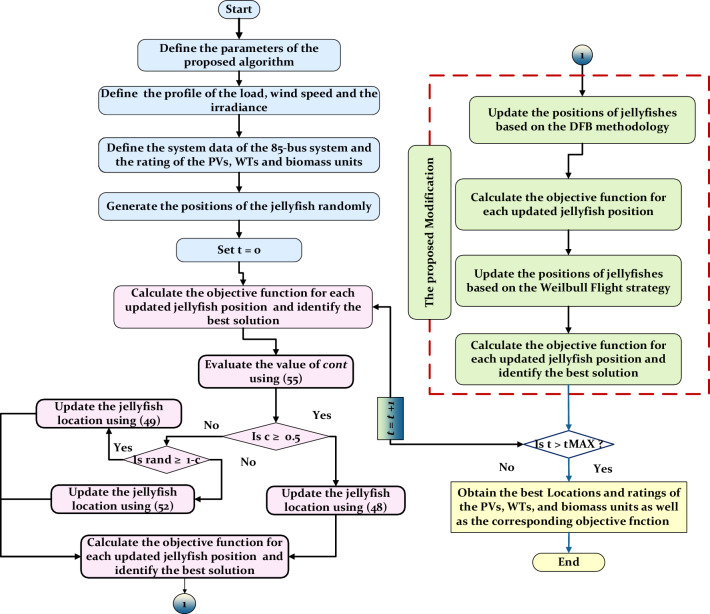
The schematic flow chart of the EJSO.

The populations’ scores in the FDB are calculated based on the normalized distance and the normalized objective function as follows:

63 $${S}_{{P}_{i}}=w\times norm{F}_{i}+(1-w)\times \text{norm}{DP}_{i}$$

where $w$ represents a weight parameter in the range 0 and 1. The flow chart of the proposed algorithm is shown in Fig. 6. It should be highlighted here that the objective function is calculated after updating the position of the population to assign the best solution and keep the best-updated population because the FDB strategy is based on the distance between the population and the best solution. Thus, it is mandatory to assign the best solution after updating the populations. The pseudocode of the proposed optimizer can be described in Algorithm 1

**Algorithm 1:**
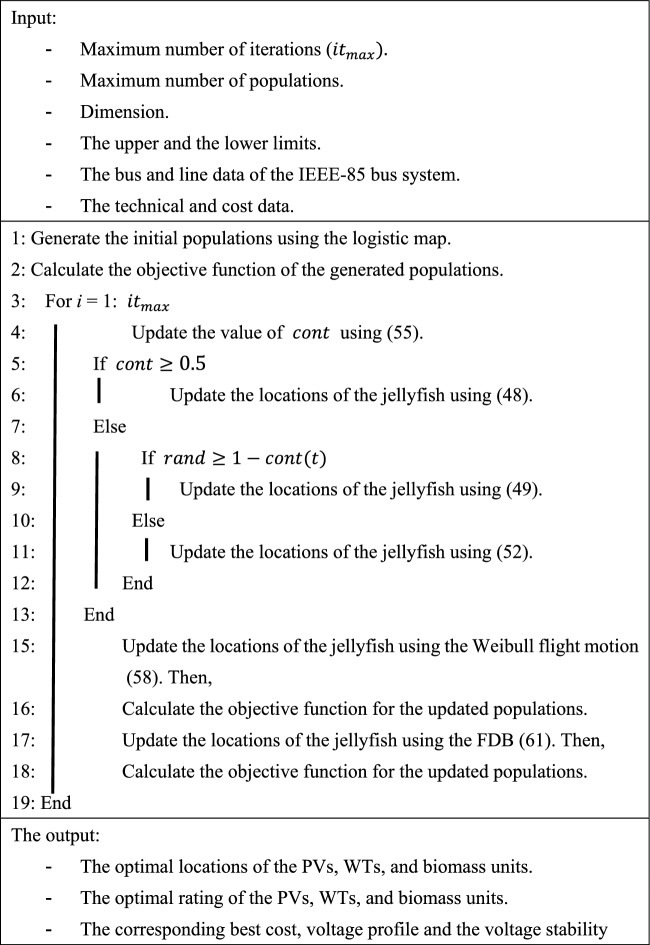
The pseudocode of EJSO.

.

## Simulation results

The suggested EJSO is employed for the EM solution of the MMGs. The obtained results have been compared to well-known other optimizers the grey wolf optimizer GWO^58^, whale optimization algorithm (WOA)^59^, sand cat search optimizer (SCSO)^60^, dandelion optimizer (DO)^61^ and Jellyfish search optimizer (JSO)^52^. The EJSO was written by the MATLAB software and the simulations were conducted on a PC with Intel i5, 4 GB RAM, 2.5 GHz CPU.

### Solving of the standard and CEC-2019 benchmark functions

In this section, the suggested EJSO has been applied to the 33 benchmark functions in which F1 to F 23 are the standard functions while CEC 01 to CEC10 are the CEC 2019 functions. The parameters of optimization methods are provided in Table 2. The standard functions including the unimodal, multimodal, and fixed-dimension benchmark functions have been described in Tables 3, 4 and 5^11,62,63^, respectively while the description of CEC 2019 functions have been depicted in Table 6^64^. The results were presented over 25 run trails.

**Table 2:** The parameters of the optimization methods.

Method	Parameter	Value
SCSO^60^	Population numbers	30
	${it}_{max}$	250
	Range of sensitivity (rg)	[2,0]
	Range of phases control (R)	[− 2rg, 2rg]
WOA^59^	Population numbers	30
	${it}_{max}$	250
	a1	[2,0]
	a2	[2, 0]
	C	2. rand (0,1)
	l	[− 1,1]
	b	1
GWO^58^	Population numbers	25
	${it}_{max}$	250
	a	[2, 0]
	A	[2, 0]
	c	2
DO^61^	Population numbers	25
	${it}_{max}$	250
	$$\alpha$$	[0,1]
	$$k$$	[0,1]
JSO^52^	Population numbers	30
	${it}_{max}$	250
	c	0.5
	$$\upgamma$$	[0.05 1]
EJSO	Population numbers	25
	${it}_{max}$	250
	c	0.5
	$w$	0.5

**Table 3:** Unimodal functions.

Function	Range	${F}_{min}$
${f}_{1}\left(h\right)={\sum }_{j=1}^{n}{h}_{j}^{2}$	[− 100, 100]	0
${f}_{2}\left(h\right)={\sum }_{j=1}^{n}\left|{h}_{j}\right|+\prod_{j=1}^{n}\left|{h}_{j}\right|$	[− 10, 10]	0
${f}_{3}\left(h\right)={\sum }_{j=1}^{n}{\left({\sum }_{i-1}^{j}{h}_{i}\right)}^{2}$	[− 100, 100]	0
${f}_{4}\left(h\right)={max}_{j} \left|{h}_{j}\right|, 1\le j\le n$	[− 100, 100]	0
${f}_{5}\left(h\right)={\sum }_{j=1}^{n-1}[100{\left({m}_{j+1}-{h}_{j}^{2}\right)}^{2}+{\left({h}_{j}-1\right)}^{2}]$	[− 30, 30]	0
${f}_{6}\left(h\right)={\sum }_{j=1}^{n-1}{\left([{h}_{j}+0.5]\right)}^{2}$	[− 100, 100]	0
${f}_{7}\left(h\right)={\sum }_{j=1}^{n}{h}_{j}^{4}+random(\text{0,1})$	[− 1.28, 1.28]	0

**Table 4:** Multimodal functions.

Function	Range	${F}_{min}$
${f}_{8}\left(k\right)={\sum }_{j=1}^{n}{- k}_{j} sin\left(\sqrt{\left|{k}_{j}\right|}\right)$	[− 500, 500]	− 418.9829*5
${f}_{9}\left(k\right)={\sum }_{j=1}^{n}\left[{k}_{j}^{2}-10 cos({2\pi k}_{j}+10)\right]$	[− 5.12, 5.12]	0
${f}_{10}\left(k\right)=-20exp\left(-0.2\sqrt{\frac{1}{n}} {\sum }_{j=1}^{n}{k}_{j}^{2}\right)-exp\left(\frac{1}{n}{\sum }_{j=1}^{n}cos({2\pi k}_{j})+20+e\right)$	[− 32, 32]	0
${f}_{11}\left(k\right)=\frac{1}{4000}{\sum }_{j=1}^{n}{k}_{j}^{2}-\prod_{j=1}^{n}cos\left(\frac{{k}_{j}}{\sqrt{j}}\right)+1$	[− 600, 600]	0
${f}_{12}\left(k\right)=\frac{\pi }{n}\left\{10\mathit{sin}\left(\pi {z}_{1}\right)+{\sum }_{j=1}^{n-1}{\left({z}_{j}-1\right)}^{2}\left[1+10{\mathit{sin}}^{2}\left(\pi {z}_{j+1}\right)\right]+{\left({z}_{n}-1\right)}^{2}\right\}+{\sum }_{j=1}^{n}u({k}_{j},10, 100, 4)$ ${z}_{j}=1+\frac{{k}_{j}+1}{4}$	[− 50, 50]	0
$$u({k}_{j},v,s,h)=\left\{\begin{array}{l}{x\left({k}_{j}-v\right)}^{h} {k}_{j}>v \\ 0 -v< {k}_{j}<v\\ {x\left(-{k}_{j}-v\right)}^{h} {k}_{j}<-v\end{array}\right.$$		
$${f}_{13}\left(k\right)=0.1\left\{{\mathit{sin}}^{2}\left(3\pi {h}_{1}\right)+{\sum }_{j=1}^{n}{\left({k}_{j}-1\right)}^{2}\left[1+{\mathit{sin}}^{2}\left(3\pi {h}_{j}+1\right)\right]+{\left({k}_{n}-1\right)}^{2}\left[1+{\mathit{sin}}^{2}\left(2\pi {h}_{j}\right)\right]\right\}+{\sum }_{j=1}^{n}u({h}_{j},5, 100, 4)$$	[− 50, 50]	
${f}_{14}\left(k\right)=-{\sum }_{j=1}^{n}\mathit{sin}\left({h}_{j}\right).\left(sin {\left(\frac{j.h}{\pi }\right)}^{2h}\right) , h=10$	$[0, \pi ]$	− 4.687
${f}_{15}\left(k\right)=\left[{e}^{-{\sum }_{j=1}^{n}{\left(\frac{{k}_{j}}{\beta }\right)}^{2h}}-2{e}^{-{\sum }_{j=1}^{n}{k}_{j}^{2}}\right]-\prod_{j=1}^{n}{cos}^{2}{ k}_{j} , h=5$	[− 20, 20]	− 1
${f}_{16}\left(k\right)=\left\{\left[{\sum }_{j=1}^{n}{sin}^{2}{ (k}_{j})\right]-exp\left(-{\sum }_{j=1}^{n}{k}_{j}^{2}\right)\right\}.exp\left[-{\sum }_{j=1}^{n}{sin}^{2}\sqrt{\left|{k}_{j}\right|}\right]$	[− 10, 10]	− 1

**Table 5:** Fixed dimension functions.

Function	Dim	Range	${F}_{min}$
${f}_{14}\left(h\right)=\frac{1}{500}+{\sum }_{i=1}^{25}\frac{1}{i+{\sum }_{j=1}^{2}{({h}_{j}-{a}_{ji})}^{6}}$	2	[− 65, 65]	1
${f}_{15}\left(h\right)={\sum }_{j=1}^{11}{\left[{ b}_{j}-\frac{{h}_{j}({b}_{j}^{2}+{b}_{j}{h}_{2})}{{b}_{j}^{2}+{b}_{j}{h}_{3+}{h}_{4}}\right]}^{2}$	4	[− 5, 5]	0.00030
${f}_{16}\left(k\right)={4h}_{1}^{2}-2.1{h}_{1}^{4}+\frac{1}{3}{h}_{1}^{6}+{h}_{1}{h}_{2}-4{h}_{2}^{2}+4{h}_{2}^{4}$	2	[− 5, 5]	− 1.0316
${f}_{17}\left(k\right)={\left({h}_{2}-\frac{5.1}{4{\pi }^{2}}{k}_{1}^{2}+\frac{5}{\pi }{h}_{1}-6\right)}^{2}+10\left(1-\frac{1}{8\pi }\right)cos{ h}_{1}+10$	2	[− 5, 5]	0.398
${f}_{18}\left(h\right)=\left[1+{\left({h}_{1}+{h}_{2}+1\right)}^{2}(19-14{h}_{1}+ {3h}_{1}^{2}-14{h}_{2}+6{h}_{1}{h}_{2}+3{h}_{2}^{2 }\right]*\left[30+{\left(2{h}_{1}-3{h}_{2}\right)}^{2}(18-32{k}_{1}+ {12h}_{1}^{2}-48{k}_{2}+36{h}_{1}{h}_{2}+27{h}_{2}^{2}\right]$	2	[− 2, 2]	3
${f}_{19}\left(h\right)=-{\sum }_{j=1}^{4}{{c}_{j} exp (-{\sum }_{i=1}^{3}{a}_{ji}\left({h}_{i}-{p}_{ji}\right)}^{2})$	3	[1, 3]	− 3.86
${f}_{20}\left(h\right)=-{\sum }_{j=1}^{4}{{c}_{j} exp (-{\sum }_{i=1}^{6}{a}_{ji}\left({h}_{i}-{p}_{ji}\right)}^{2})$	6	[0, 1]	− 3.32
${f}_{21}\left(h\right)=-{\sum }_{j=1}^{5}{\left[\left(k-{a}_{j}\right){\left(k-{a}_{j}\right)}^{T}+{c}_{j}\right]}^{-1} )$	4	[0, 10]	− 10.1532
${f}_{22}\left(h\right)=-{\sum }_{j=1}^{7}{\left[\left(h-{a}_{j}\right){\left(h-{a}_{j}\right)}^{T}+{c}_{j}\right]}^{-1} )$	4	[0, 10]	− 10.4028
${f}_{23}\left(h\right)=-{\sum }_{j=1}^{10}{\left[\left(h-{a}_{j}\right){\left(h-{a}_{j}\right)}^{T}+{c}_{j}\right]}^{-1} )$	4	[0, 10]	− 10.5363

**Table 6:** The CEC 2019 functions.

Function	Optimal fitness	Boundaries
$$\begin{array}{l}{Fu.}_{\text{CEC}1}={f}_{1}+{f}_{2}+{f}_{3}\\ {f}_{1}=\left\{\begin{array}{ll}(u-d{)}^{2} & \quad {\text{i}}{\text{f}} \, u < d,\\ 0 & \quad {\text{otherwise}}; \end{array}u=\sum_{j=1}^{D} {x}_{j}(1.2{)}^{D-j}\right.\\ {f}_{2}=\left\{\begin{array}{ll}(v-d{)}^{2} & \quad {\text{i}}{\text{f}} \, v < d,v=\sum_{j=1}^{D} {x}_{j}(-1.2{)}^{D-j}\\ 0 & \quad {\text{otherwise}} ; \end{array}\right.\\ {f}_{k}=\left\{\begin{array}{ll}{\left({w}_{k}-1\right)}^{2} & \quad {\text{i}}{\text{f}} \, {w}_{k} > 1\\ {\left({w}_{k}+1\right)}^{2} & \quad {\text{i}}{\text{f}} \, {w}_{k} < 1\\ 0 & \quad {\text{otherwise}} ; \end{array}{w}_{k}=\sum_{j=1}^{D} {x}_{j}{\left(\frac{2k}{m}-1\right)}^{D-j}\right.\\ {f}_{3}=\sum_{k=0}^{m} {p}_{k},k=\text{0,1},\dots ,m,m=32Dd=72.661 \; {\text{for}} \; D=9\end{array}$$	1	[− 8192 8192]
${Fu.}_{\text{CEC}2}=\sum_{i=1}^{n} \sum_{k=1}^{n} \left|{w}_{i,k}\right|$ $$\left({w}_{i,k}\right)=\textbf{W}=\textbf{H}\textbf{Z}-\textbf{I},\textbf{I}=\left[\begin{array}{cccc}1& 0& \dots & 0\\ 0& 1& \dots & 0\\ \vdots & \vdots & \ddots & \vdots \\ 0& 0& \dots & 1\end{array}\right]$$ $\textbf{H}=\left({h}_{i,k}\right),{h}_{i,k}=\frac{1}{i+k-1},i,k=\text{1,2},\dots ,n,n={\sqrt{D}}^{-}$ $\textbf{Z}=\left({z}_{i,k}\right),{z}_{i,k}={x}_{i+n(k-1)}$	1	[− 16384 16384]
$$\begin{array}{l}{Fu.}_{\text{CEC}3}=12.7120622568+\sum_{i=1}^{n-1} \sum_{j=i+1}^{n} \left(\frac{1}{{d}_{i,j}^{2}}-\frac{2}{{d}_{i,j}}\right),\\ {d}_{i,j}={\left(\sum_{k=0}^{2} {\left({x}_{3i+k-2}-{x}_{3j+k-2}\right)}^{2}\right)}^{3},n=D/3\end{array}$$	1	[− 4 4]
${Fu.}_{\text{CEC}4}=\sum_{i=1}^{D} \left({x}_{i}^{2}-10\text{cos}\left(2\pi {x}_{i}\right)+11\right)$	1	[− 100 100]
${Fu.}_{\text{CEC}5}=\sum_{i=1}^{D} \frac{{x}_{i}^{2}}{4000}-\prod_{i=1}^{D} \text{cos}\left(\frac{{x}_{i}}{\sqrt{i}}\right)+1$	1	[− 100 100]
${Fu.}_{\text{CEC}6}=\sum_{i=1}^{D} \left(\sum_{k=0}^{{k}_{max}} \left[{a}^{k}\text{cos}\left(2\pi {b}^{k}\left({x}_{i}+0.5\right)\right)\right]\right)-D\sum_{k=0}^{{k}_{max}} {a}^{k}\text{cos}\left(\pi {b}^{k}\right)$ $a=0.5,b=3,{k}_{max}=20$	1	[− 100 100]
$$\begin{array}{l}\begin{array}{l}{Fu.}_{\text{CEC}7}=418.9829D-\sum_{i=1}^{D} g\left({z}_{i}\right)\\ {z}_{i}={x}_{i}+420.9687462275036\end{array}\\ g\left({z}_{i}\right)=\left\{\begin{array}{c}{z}_{i}sin\left({\left|{z}_{i}\right|}^{1/2}\right)\\ \left(500-mod\left({z}_{i},500\right)\right)sin\left(\sqrt{\left|500-mod\left({z}_{i},500\right)\right|}\right)\\ \left(mod\left(\left|{z}_{i}\right|,500\right)-500\right)sin\left(\sqrt{\left|mod\left({z}_{i},500\right)-500\right|}\right)\end{array}\right.\end{array}$$	1	[− 100 100]
$$\begin{array}{l}{Fu.}_{\text{CEC}8}=g\left({x}_{1},{x}_{2}\right)+g\left({x}_{2},{x}_{3}\right)\dots +g\left({x}_{D-1},{x}_{D}\right)+g\left({x}_{D},{x}_{1}\right)\\ g(x,y)=0.5+\frac{{\text{sin}}^{2}\left(\sqrt{{x}^{2}+{y}^{2}}\right)-0.5}{{\left(1+0.001\left({x}^{2}+{y}^{2}\right)\right)}^{2}}\end{array}$$	1	[− 100 100]
${Fu.}_{\text{CEC}9}={\left|\sum_{i=1}^{D} {x}_{i}^{2}-D\right|}^\frac{1}{4}+\frac{\left(0.5\sum_{i=1}^{D} {x}_{i}^{2}+\sum_{i=1}^{D} {x}_{i}\right)}{D}+0.5$	1	[− 100 100]
$$\begin{array}{l}\\ {Fu.}_{\text{CEC}10}=-20exp\left(0.2\sqrt{\frac{1}{D}\sum_{i=1}^{D} {x}_{i}^{2}}\right)\\ -exp\left(\frac{1}{D}\sum_{i=1}^{D} \text{cos}\left(2\pi {x}_{i}\right)\right)+20+e\end{array}$$	1	[− 100 100]

#### Analysis of the statistical results

Here, the performance of EJSO has been compared with GWO, DO, WOA, SCSO, and the standard JSO in terms of the worst, the best, the mean, the Wilcoxon p-values, and the standard deviation (SD) values as depicted in Table 7 for the standard and the CEC-2019 functions. The bolded values in this table refer to the best statistical results. As per the results of Table 7, the proposed EJSO algorithm has superior results in the most objective functions. However, the results are similar with SCSO for F11, JSO for F14, all optimizers for F16, all optimizers except WOA for F17, DO and JSO for CEC02, and all optimizers for CEC03. Furthermore, some optimizers give results better than the EJSO like SCSO for F10 and DO for CEC01, DO for CEC10. According to the Wilcoxon test, the p-values are less than 0.05 which verified there is a notable difference between the obtained results from the suggested algorithm and the other optimization methods. The *p*-values for F9, F11, and F17 are not available, this means that the results are identical for all trail runs. The computational time for SCSO, GWO, WOA, DO, JSO, and EJSO algorithms are 61.4, 91.2, 150.8, 208.6, 368.3 and 1780.6 respectively. It should be highlighted here that the computational time of the proposed algorithm EJSO is slightly more than the original algorithm, this is due to the added modifications to the original algorithm. However, the EJSO needs more time, but the accuracy of the obtained results is the best.

**Table 7:** The statistical outcomes of the standard and CEC 2019 functions.

Fun	Algorithms	Mean	Best	Worst	SD	P-value
F1	SCSO	2.7275E−53	2.8051E−61	6.7149E−52	1.3422E−52	1.4157E−09
	GWO	8.4143E−11	3.7517E−12	3.2150E−10	8.6162E−11	1.4157E−09
	WOA	3.2804E−33	4.2420E−40	7.3260E−32	1.4600E−32	1.4157E−09
	DO	8.2000E−3	3.2000E−3	1.8100E−2	4.4000E−3	1.4157E−09
	JSO	1.0639E−09	1.1833E−17	2.6592E−08	5.3183E−09	1.4157E−09
	EJSO	**1.1007E−85**	**6.8664E−95**	**2.7393E−84**	5.4775E−85	–
F2	SCSO	8.1121E−29	1.2420E−33	1.1483E−27	2.3280E−28	1.4157E−09
	GWO	3.3928E−07	1.4614E−07	7.5505E−07	1.3911E−07	1.4157E−09
	WOA	3.9408E−24	5.8900E−28	2.8426E−23	6.5247E−24	1.4157E−09
	DO	3.5100E−02	1.2500E−02	8.1000E−02	1.3900E−02	1.4157E−09
	JSO	5.8713E−05	1.6972E−09	1.4000E−03	2.8110E−04	1.4157E−09
	EJSO	**2.0814E−44**	**9.2086E−50**	**3.1682E−43**	6.3475E−44	–
F3	SCSO	9.1005E−46	1.6835E−54	2.0017E−44	4.0122E−45	1.4157E−09
	GWO	4.4750E−01	2.8300E−02	1.76720	4.7400E−01	1.4157E−09
	WOA	8.1218E+04	4.2435E+04	1.2485E+05	1.9389E+04	1.4157E−09
	DO	4.0318E+02	6.9377E+01	1.4826E+03	3.2045E+02	1.4157E−09
	JSO	6.0060E+01	9.6000E−03	7.7111E+02	1.6228E+02	1.4157E−09
	EJSO	**1.2636E−56**	**1.4834E−68**	**3.1201E−55**	6.2371E−56	–
F4	SCSO	7.9730E−24	4.2127E−28	1.2269E−22	2.5036E−23	1.4157E−09
	GWO	1.2100E−02	2.6000E−03	3.4100E−02	7.1000E−03	1.4157E−09
	WOA	7.1145E+01	1.7419E+01	9.0217E+01	1.8344E+01	1.4157E−09
	DO	9.72860	2.08010	2.7369E+01	6.09170	1.4157E−09
	JSO	8.7169E−09	1.3561E−09	5.7025E−08	1.1709E−08	1.4157E−09
	EJSO	**5.8937E−42**	**2.7748E−45**	**9.9598E−41**	2.0287E−41	–
F5	SCSO	2.8555E+01	2.7071E+01	2.8850E+01	5.0770E−01	1.4157E−09
	GWO	2.7701E+01	2.6407E+01	2.8840E+01	7.4120E−01	1.4157E−09
	WOA	2.8633E+01	2.8027E+01	2.8821E+01	1.6430E−01	1.4157E−09
	DO	5.9578E+01	2.5420E+01	2.2625E+02	5.2945E+01	1.4157E−09
	JSO	1.45720	1.9700E−02	1.4347E+01	2.96670	2.5677E−08
	EJSO	**9.9620E−01**	**3.2970E−06**	**2.4900E+01**	4.98000	–
F6	SCSO	2.65690	1.26970	4.25910	7.0620E−01	1.4157E−09
	GWO	1.33930	7.4580E−01	2.28380	3.3090E−01	1.4157E−09
	WOA	1.42080	3.8930E−01	2.06310	4.9880E−01	1.4157E−09
	DO	2.1000E−03	5.1005E−04	7.0000E−03	1.5000E−03	1.4157E−09
	JSO	5.0373E−05	1.4586E−06	5.3563E−04	1.0959E−04	1.4157E−09
	EJSO	**5.3181E−11**	**1.9616E−12**	**2.5064E−10**	6.4500E−11	–
F7	SCSO	**4.0512E−04**	**1.0472E−05**	**2.5000E−03**	5.6652E−04	4.1349E−04
	GWO	5.4000E−03	1.9000E−03	1.5200E−02	3.2000E−03	1.4157E−09
	WOA	7.4000E−03	1.6201E−04	2.5500E−02	6.9000E−03	5.0255E−07
	DO	5.2800E−02	2.9000E−02	1.0240E−01	1.7100E−02	1.4157E−09
	JSO	1.1000E−03	8.2861E−05	6.3000E−03	1.3000E−03	1.9360E−01
	EJSO	6.9187E−04	2.1439E−04	1.5000E−03	3.5810E−04	–
F8	SCSO	− 6.5434E+03	− 7.6880E+03	− 4.7898E+03	7.4889E+02	1.4157E−09
	GWO	− 5.7629E+03	− 7.4258E+03	− 2.6197E+03	1.0016E+03	1.4157E−09
	WOA	− 9.6917E+03	− 1.2566E+04	− 5.8162E+03	1.9222E+03	4.6094E−05
	DO	− 7.1133E+03	− 8.2335E+03	− 5.4329E+03	6.8754E+02	1.4157E−09
	JSO	− 8.2338E+03	− 1.1051E+04	− 5.6912E+03	1.5397E+03	2.8980E−09
	EJSO	**− 1.1926E+04**	**− 1.2569E+04**	**− 1.0178E+04**	7.1042E+02	–
F9	SCSO	**0.00000**	**0.00000**	**0.00000**	0.00000	N/A
	GWO	1.1719E+01	1.1981E−05	3.0952E+01	6.95840	9.7285E−11
	WOA	4.5475E−15	0.00000	5.6843E−14	1.5739E−14	1.6140E−01
	DO	4.0734E+01	6.34330	1.2993E+02	2.8498E+01	9.7285E−11
	JSO	1.6740E−01	1.9000E−03	9.5300E−01	2.7780E−01	9.7285E−11
	EJSO	**0.00000**	**0.00000**	**0.00000**	0.00000	–
F10	SCSO	**8.8818E−16**	**8.8818E−16**	**8.8818E−16**	0.00000	3.3710E−01
	GWO	1.4145E−06	6.0982E−07	4.8316E−06	9.6452E−07	1.3762E−10
	WOA	1.1831E−14	8.8818E−16	4.3521E−14	1.1231E−14	6.7484E−10
	DO	2.1540E−01	1.2400E−02	1.65140	4.3900E−01	1.3762E−10
	JSO	4.3903E−07	1.2681E−09	6.1150E−06	1.2948E−06	1.3762E−10
	EJSO	1.0303E−15	8.8818E−16	4.4409E−15	7.1054E−16	–
F11	SCSO	**0.00000**	**0.00000**	**0.00000**	0.00000	N/A
	GWO	5.2000E−03	8.6030E−12	3.7700E−02	1.0300E−02	9.7285E−11
	WOA	1.6600E−02	0.00000	4.1600E−01	8.3200E−02	3.3710E−01
	DO	3.5100E−02	5.4000E−03	9.7900E−02	1.9900E−02	9.7285E−11
	JSO	3.1796E−12	0.00000	4.9023E−11	1.1316E−11	2.4574E−04
	EJSO	**0.00000**	**0.00000**	**0.00000**	0.00000	–
F12	SCSO	1.6120E−01	5.9600E−02	4.4350E−01	7.8700E−02	1.4157E−09
	GWO	9.6100E−02	3.3800E−02	2.7010E−01	5.5300E−02	1.4157E−09
	WOA	9.9000E−02	3.0800E−02	2.0910E−01	5.4300E−02	1.4157E−09
	DO	4.3620E−01	2.1374E−05	2.81400	6.8150E−01	1.4157E−09
	JSO	5.4011E−07	1.7945E−08	2.6155E−06	5.7871E−07	1.4157E−09
	EJSO	**9.7064E−13**	**3.0439E−14**	**4.3430E−12**	1.1550E−12	1.4157E−09
F13	SCSO	2.71630	2.06620	2.97960	1.9540E−01	1.4157E−09
	GWO	9.8660E−01	4.4710E−01	1.48740	2.8930E−01	1.4157E−09
	WOA	1.10370	4.6500E−01	1.85140	3.3200E−01	1.4157E−09
	DO	3.3660E−01	4.8706E−04	7.24750	1.44140	1.4157E−09
	JSO	4.8289E−04	1.4660E−06	1.1800E−02	2.4000E−03	1.4157E−09
	EJSO	**3.4772E−11**	**4.5481E−13**	**6.2433E−10**	1.2307E−10	–
F14	SCSO	3.97970	9.9800E−1	1.2671E+1	3.7786	9.7285E−11
	GWO	4.40960	9.9800E−1	1.2671E+01	3.95880	9.7285E−11
	WOA	3.54760	9.9800E−01	1.5504E+01	3.68890	9.7285E−11
	DO	1.51300	9.9800E−01	5.92880	1.10880	9.6829E−11
	JSO	**9.98E−1**	**9.98E−1**	**9.98E−1**	1.7554E−16	9.2584E−05
	EJSO	**9.98E−1**	**9.98E−1**	**9.98E−1**	0.00000	–
F15	SCSO	4.6449E−04	3.0749E−04	1.2000E−03	1.9961E−04	1.3641E−09
	GWO	5.6000E−03	3.0969E−04	2.0400E−02	8.6000E−03	1.3641E−09
	WOA	7.0728E−04	3.1175E−04	2.3000E−03	5.1214E−04	1.3641E−09
	DO	3.7000E−03	3.0766E−04	2.0400E−02	7.4000E−03	1.3641E−09
	JSO	3.1172E−04	3.0749E−04	3.9780E−04	1.7998E−05	1.3641E−09
	EJSO	**3.0749E−4**	**3.0749E−4**	**3.0749E−4**	1.8081E−19	–
F16	SCSO	**− 1.03160**	**− 1.03160**	**− 1.03160**	7.2179E−09	3.8499E−10
	GWO	**− 1.03160**	**− 1.03160**	**− 1.03160**	1.0750E−07	3.8499E−10
	WOA	**− 1.03160**	**− 1.03160**	**− 1.03160**	8.4348E−08	3.8499E−10
	DO	**− 1.03160**	**− 1.03160**	**− 1.03160**	5.3660E−12	3.8499E−10
	JSO	**− 1.03160**	**− 1.03160**	**− 1.03160**	5.7332E−16	2.0300E−02
	EJSO	**− 1.03160**	**− 1.03160**	**− 1.03160**	6.4099E−16	–
F17	SCSO	**3.9790E−1**	**3.9790E−1**	**3.9790E−1**	1.2146E−07	9.7285E−11
	GWO	**3.9790E−1**	**3.9790E−1**	**3.9790E−1**	2.1425E−06	9.7285E−11
	WOA	3.9800E−1	3.9790E−1	3.9830E−1	1.1302E−04	9.7285E−11
	DO	**3.9790E−1**	**3.9790E−1**	**3.9790E−1**	3.3212E−10	9.7285E−11
	JSO	**3.9790E−1**	**3.9790E−1**	**3.9790E−1**	0.00000	N/A
	EJSO	**3.9790E−1**	**3.9790E−1**	**3.9790E−1**	0.00000	–
F18	SCSO	3.00010	3.00000	3.00040	9.5900E−05	5.6367E−10
	GWO	3.00020	3.00000	3.00080	1.9779E−04	5.6367E−10
	WOA	7.32850	3.00000	3.0113E+01	1.0121E+01	5.6367E−10
	DO	9.48000	3.00000	8.4000E+01	2.2428E+01	5.6367E−10
	JSO	**3.00000**	**3.00000**	**3.00000**	1.5622E−15	–
	EJSO	**3.00000**	**3.00000**	**3.00000**	1.0415E−15	5.6367E−10
F19	SCSO	− 3.85950	− 3.86280	− 3.85490	3.7000E−03	9.7285E−11
	GWO	− 3.86050	− 3.86280	− 3.85490	2.9000E−03	9.7285E−11
	WOA	− 3.77550	− 3.86270	− 3.07570	1.7030E−01	9.7285E−11
	DO	**− 3.86280**	**− 3.86280**	**− 3.86280**	1.9504E−06	9.7285E−11
	JSO	**− 3.86280**	**− 3.86280**	**− 3.86280**	2.0431E−15	3.5268E−05
	EJSO	**− 3.86280**	**− 3.86280**	**− 3.86280**	2.2662E−15	–
F20	SCSO	− 3.22960	− 3.32200	− 3.08670	7.6100E−02	1.9275E−09
	GWO	− 3.25680	− 3.32200	− 3.09090	7.8800E−02	6.6476E−09
	WOA	− 3.18310	− 3.31990	− 2.81780	1.3250E−01	4.0711E−09
	DO	− 3.27920	− 3.32200	− 3.20300	5.8300E−02	1.0784E−08
	JSO	− 3.32200	− 3.32200	− 3.32200	1.7864E−09	8.7795E−08
	EJSO	**− 3.31250**	**− 3.32200**	**− 3.20310**	3.2900E−02	–
F21	SCSO	− 5.27670	− 1.0153E+1	− 8.8100E−01	2.58840	7.5434E−10
	GWO	− 9.14170	− 1.0152E+1	− 2.62970	2.39830	7.5434E−10
	WOA	− 7.61240	− 1.0147E+1	− 5.03730	2.51610	7.5434E−10
	DO	− 7.03060	− 1.0153E+1	− 2.63050	3.18340	7.5434E−10
	JSO	− 1.0129E+1	− 1.0153E+1	− 9.82130	6.9900E−02	7.5434E−10
	EJSO	**− 1.0153E+01**	**− 1.0153E+01**	**− 1.0153E+01**	4.7277E−15	–
F22	SCSO	− 6.38070	− 1.0403E+01	− 9.0810E−01	3.60010	6.0586E−10
	GWO	− 1.0183E+01	− 1.0402E+01	− 5.12040	1.05460	6.0586E−10
	WOA	− 6.89230	− 1.0380E+01	− 2.72780	3.07880	6.0586E−10
	DO	− 6.92540	− 1.0403E+01	− 1.83760	3.50130	6.0586E−10
	JSO	**− 1.0403E+1**	**− 1.0403E+1**	**− 1.0403E+1**	1.5348E−05	1.5048E−09
	EJSO	**− 1.0403E+1**	**− 1.0403E+1**	**− 1.0403E+1**	3.1611E−15	–
F23	SCSO	− 6.91180	− 1.0536E+01	− 1.67650	3.16260	2.4606E−10
	GWO	− 1.0099E+01	− 1.0535E+01	− 5.12830	1.48940	2.4606E−10
	WOA	− 5.85920	− 1.0439E+01	− 1.66300	2.85680	2.4606E−10
	DO	− 5.76080	− 1.0536E+01	− 1.67660	3.76480	2.4606E−10
	JSO	**− 1.0536E+01**	**− 1.0536E+01**	**− 1.0536E+01**	7.8504E−05	4.2863E−10
	EJSO	**− 1.0536E+01**	**− 1.0536E+01**	**− 1.0536E+01**	1.7007E−15	–
CEC01	SCSO	4.6954E+04	4.0723E+04	6.3404E+04	5.2221E+03	2.2967E−08
	GWO	9.0113E+08	1.1120E+06	5.7758E+09	1.4482E+09	1.4157E−09
	WOA	4.8776E+10	7.3342E+06	1.8118E+11	4.5772E+10	1.4157E−09
	DO	1.2377E+09	4.9996E+06	5.4237E+09	1.3986E+09	1.4157E−09
	JSO	1.2525E+08	1.8745E+06	1.0246E+09	2.3207E+08	1.4157E−09
	EJSO	**3.8005E+04**	**3.3570E+04**	**4.6743E+04**	2.8006E+03	–
CEC02	SCSO	1.8395E+01	1.8343E+01	1.8708E+01	1.1950E−01	1.3762E−10
	GWO	1.8345E+01	1.8344E+01	1.8346E+01	6.5801E−04	1.3762E−10
	WOA	1.8423E+01	1.8346E+01	1.8681E+01	8.8500E−02	1.3762E−10
	DO	**1.8343E+01**	**1.8343E+01**	**1.8343E+01**	5.6652E−05	1.3762E−10
	JSO	**1.8343E+01**	**1.8343E+01**	**1.8343E+01**	4.2456E−10	1.3762E−10
	EJSO	**1.8343E+01**	**1.8343E+01**	**1.8343E+01**	7.1423E−15	–
CEC03	SCSO	1.37030E+1	1.3702E+1	1.3704E+01	3.7308E−04	6.5661E−10
	GWO	**1.3702E+1**	**1.3702E+1**	**1.3702E+1**	8.8487E−06	6.5661E−10
	WOA	**1.3702E+1**	**1.3702E+1**	**1.3702E+1**	3.5753E−06	6.5661E−10
	DO	**1.3702E+1**	**1.3702E+1**	**1.3702E+1**	2.2921E−10	6.5661E−10
	JSO	**1.3702E+1**	**1.3702E+1**	**1.3702E+1**	9.0260E−10	–
	EJSO	**1.3702E+1**	**1.3702E+1**	**1.3702E+1**	2.0041E−14	6.5661E−10
CEC04	SCSO	1.1813E+03	5.4198E+01	4.8614E+03	1.3034E+03	1.4157E−09
	GWO	1.7482E+02	4.8579E+01	2.4705E+03	4.7877E+02	1.8002E−09
	WOA	9.9568E+02	3.2253E+02	2.5192E+03	6.4843E+02	1.4157E−09
	DO	5.5684E+01	1.4015E+01	1.7256E+02	3.2425E+01	2.8526E−04
	JSO	1.8445E+02	5.3872E+01	9.0704E+02	1.8243E+02	1.4157E−09
	EJSO	**2.9680E+01**	**1.1946E+01**	**4.9753E+01**	1.0662E+01	–
CEC05	SCSO	2.50280	2.20680	4.07350	3.6290E−01	1.5967E−09
	GWO	2.57030	2.12570	2.92050	2.5580E−01	2.5742E−09
	WOA	3.04430	2.52000	4.20470	3.4750E−01	1.4157E−09
	DO	2.30360	2.05250	2.99190	2.3090E−01	1.3090E−07
	JSO	2.14300	2.05700	2.26130	5.8800E−02	1.4298E−04
	EJSO	**2.08420**	**2.00990**	**2.20680**	3.9400E−02	–
CEC06	SCSO	9.29800	6.64800	1.2204E+01	1.53440	3.2134E−06
	GWO	1.2497E+01	9.99700	1.4186E+01	8.4170E−01	1.4157E−09
	WOA	1.0812E+01	8.85120	1.2589E+01	1.02520	2.2857E−09
	DO	7.82560	4.49030	1.1254E+01	1.79980	2.4400E−02
	JSO	1.2267E+01	1.0273E+01	1.4005E+01	9.4420E−01	1.4157E−09
	EJSO	**6.70840**	**4.10290**	**9.19120**	1.37470	–
CEC07	SCSO	3.7211E+02	1.2258E+02	9.5967E+02	1.6998E+02	1.5967E−09
	GWO	6.2014E+02	2.7831E+01	1.2781E+03	3.1125E+02	2.8980E−09
	WOA	7.2703E+02	3.0951E+02	1.3911E+03	2.5970E+02	1.4157E−09
	DO	3.0527E+02	− 1.1300E+02	7.0535E+02	2.4187E+02	1.1153E−06
	JSO	9.3766E+02	6.5135E+02	1.1517E+03	1.6396E+02	1.4157E−09
	EJSO	**− 2.4068E+01**	**− 2.5143E+02**	**1.2594E+02**	8.7525E+01	–
CEC08	SCSO	5.62600	4.24860	6.87150	6.9070E−01	1.9933E−07
	GWO	5.60510	3.87950	7.05130	8.1640E−01	6.1473E−07
	WOA	6.22490	4.92950	7.18380	5.0800E−01	2.0288E−09
	DO	5.35370	3.92920	6.16280	5.7390E−01	8.2938E−07
	JSO	5.71180	3.90810	6.64120	6.0840E−01	2.5677E−08
	EJSO	4.22420	2.93780	5.33730	6.4740E−01	–
CEC09	SCSO	5.7994E+01	4.42630	6.5593E+02	1.5405E+02	1.4157E−09
	GWO	1.6765E+01	4.03200	2.7495E+02	5.3797E+01	1.4157E−09
	WOA	2.8145E+01	5.40420	1.2262E+02	2.4971E+01	1.4157E−09
	DO	3.99840	3.45460	5.06820	4.5120E−01	6.5743E−09
	JSO	4.30450	3.64800	5.44870	4.0870E−01	1.4157E−09
	EJSO	**3.41640**	**3.35430**	**3.56390**	4.7400E−02	–
CEC10	SCSO	2.1193E+01	2.0999E+01	2.1494E+01	1.3780E−01	9.5133E−08
	GWO	2.1555E+01	2.1321E+01	2.1719E+01	1.0580E−01	1.4157E−09
	WOA	2.1399E+01	2.1156E+01	2.1618E+01	1.0800E−01	1.4157E−09
	DO	**2.0343E+01**	**2.66490**	**2.1453E+01**	3.68450	5.1234E−06
	JSO	2.1298E+01	1.4694E+01	2.1730E+01	1.38170	2.2967E−08
	EJSO	2.0364E+01	5.01720	2.1025E+01	3.19730	–

Bold values refer to the optimal obtained values compared to other values.

#### The convergence analysis

**Figure 7:**
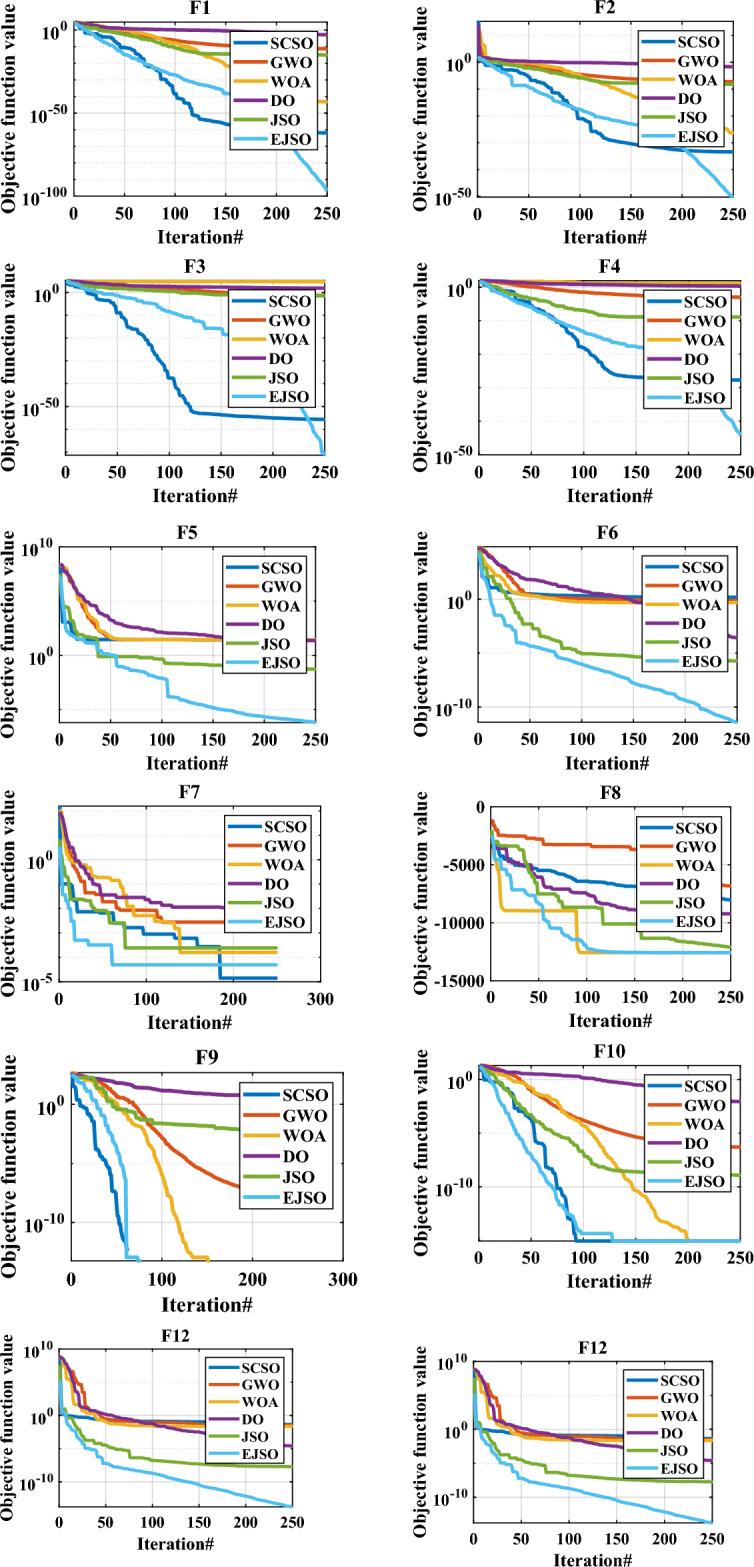

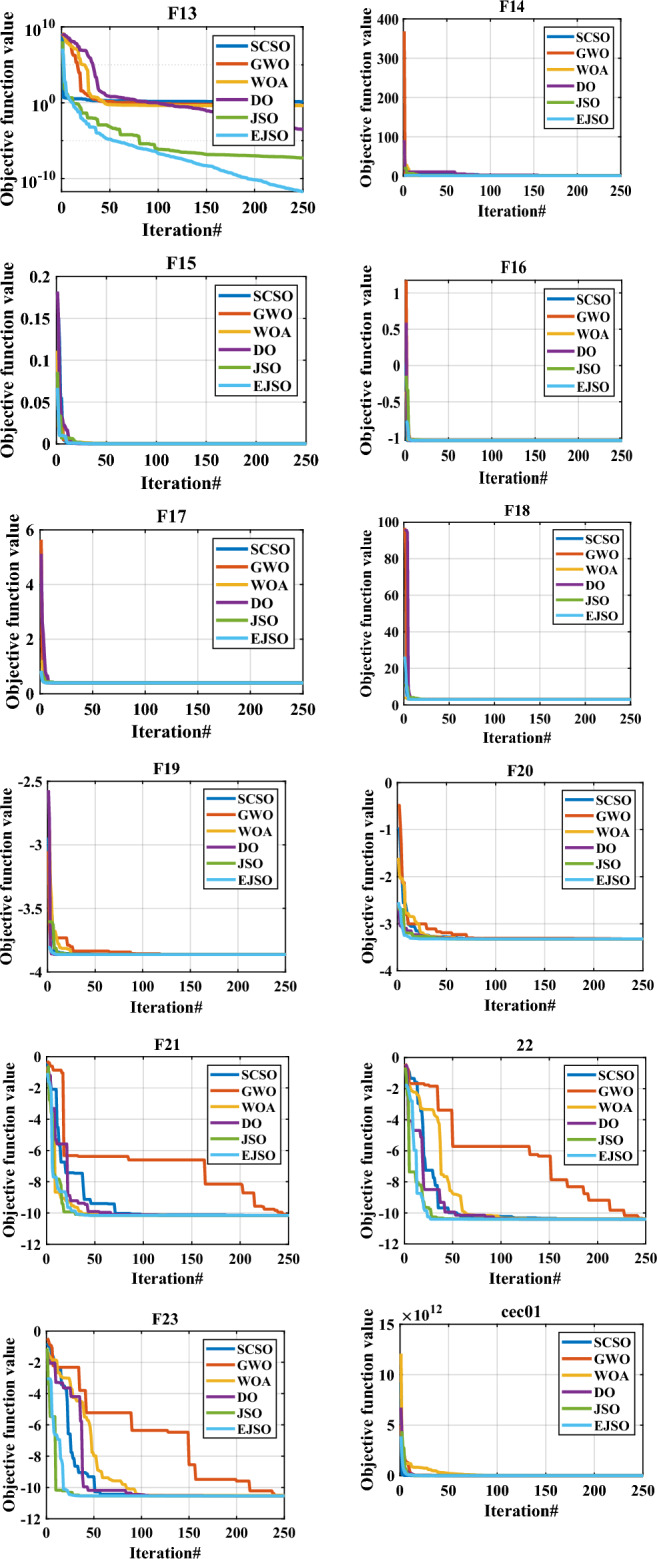

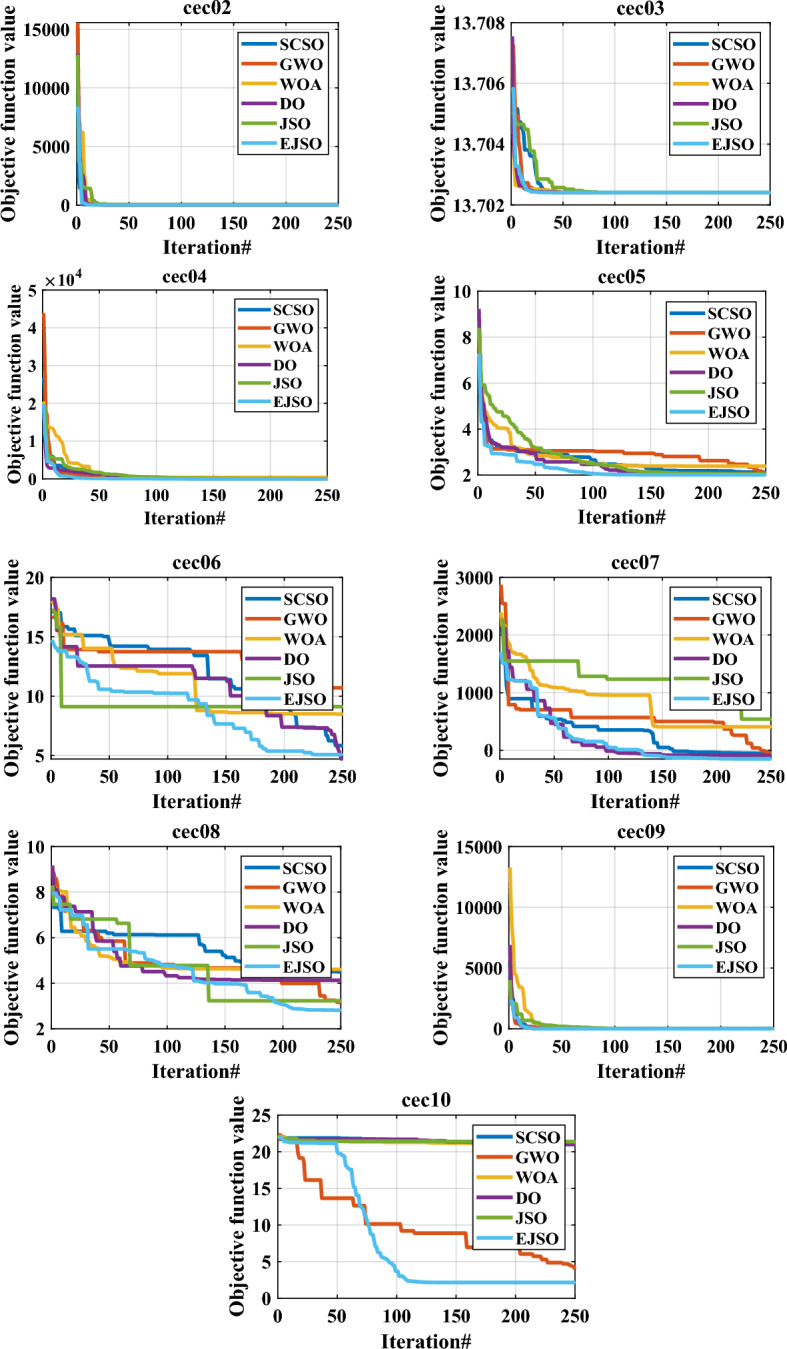
The convergence of benchmark functions by different optimizers.

The convergence characteristics of the proposed EJSO, GWO, DO, WOA, SCSO, and the standard JSO are illustrated in Fig. 7. It is evident from the convergence characteristics, that the EJSO has good convergence for the unimodal, the multimodal, the fixed-dimension, and the CEC-2019 functions. However, the convergence of the DO is the best for CEC10.

#### The analysis of the boxplot

**Figure 8:**
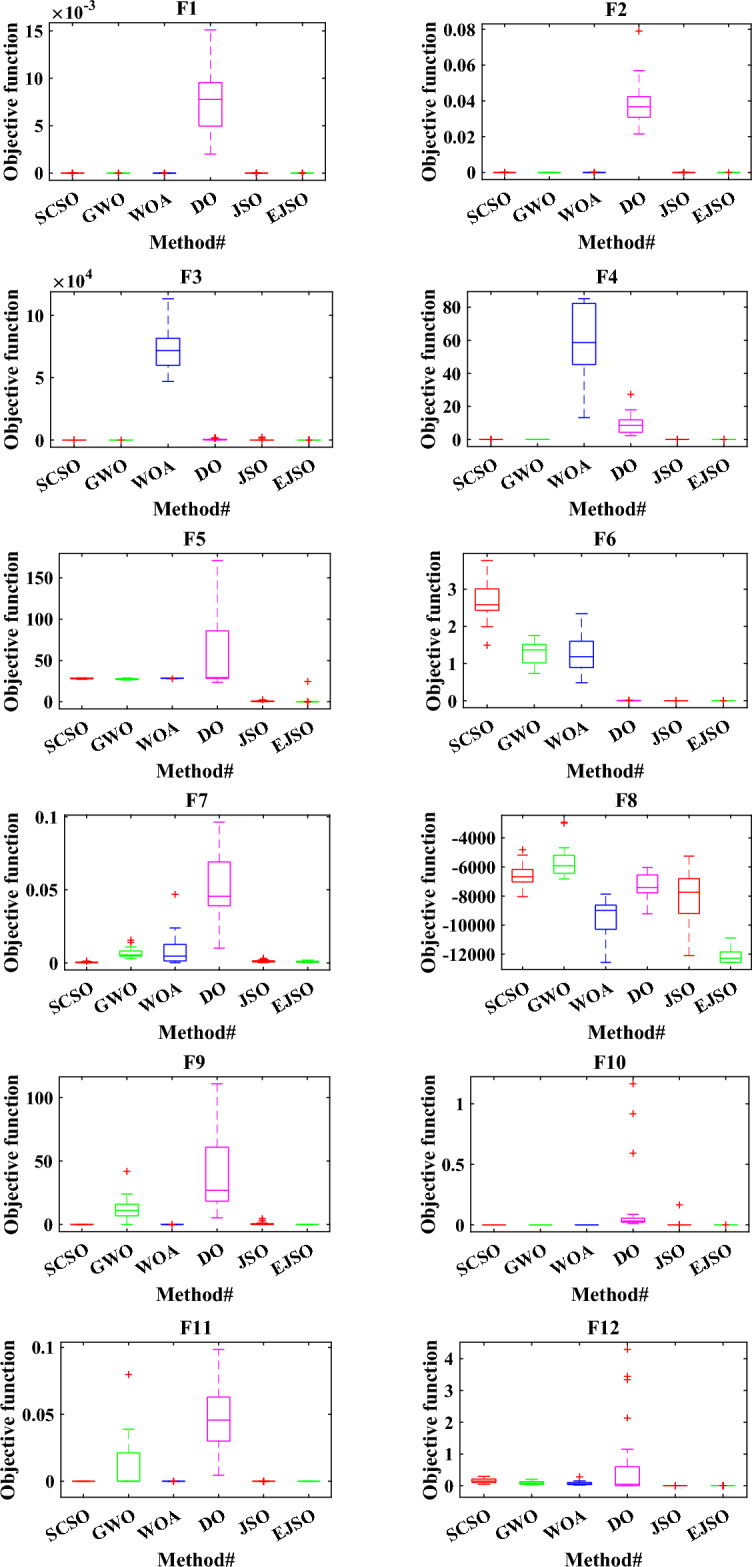

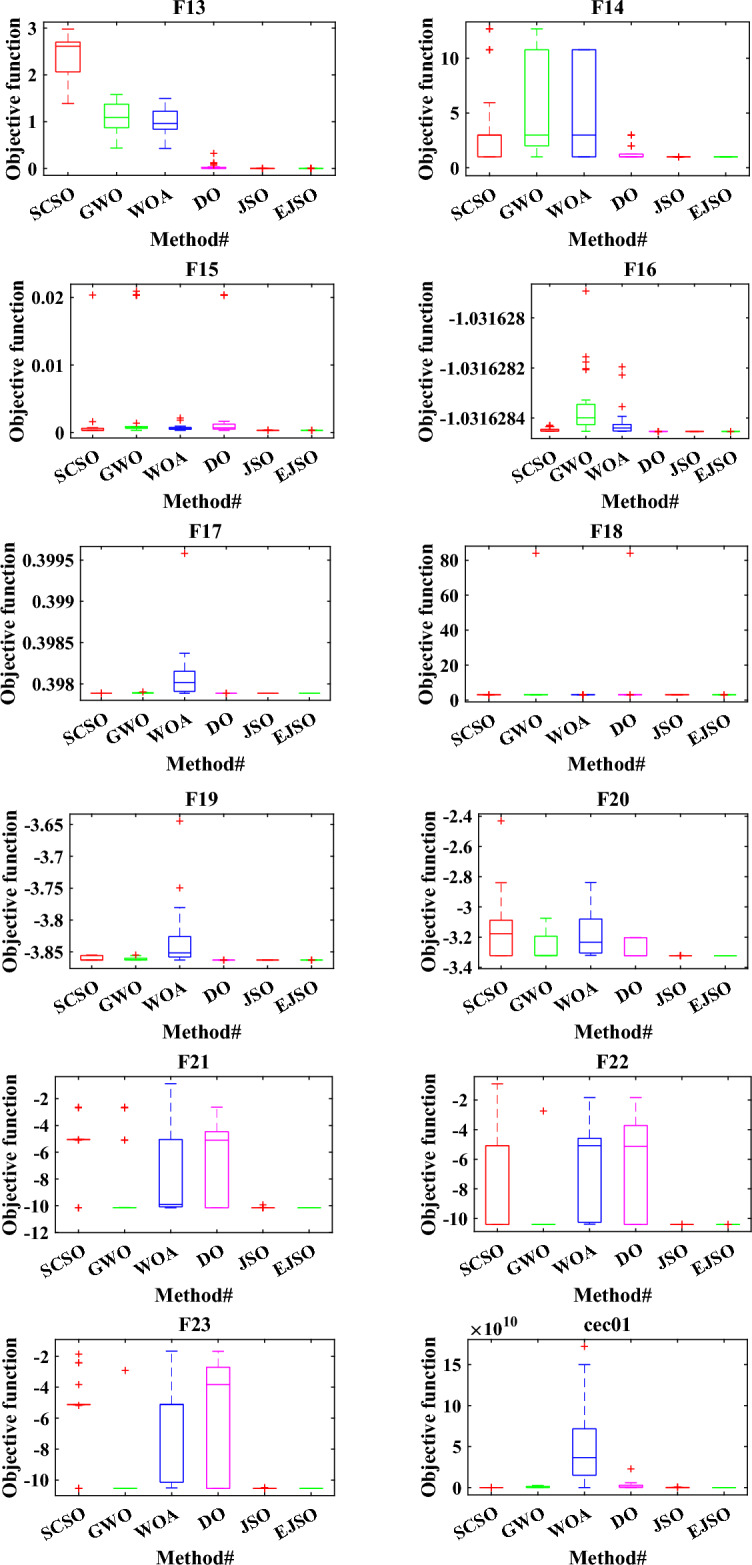

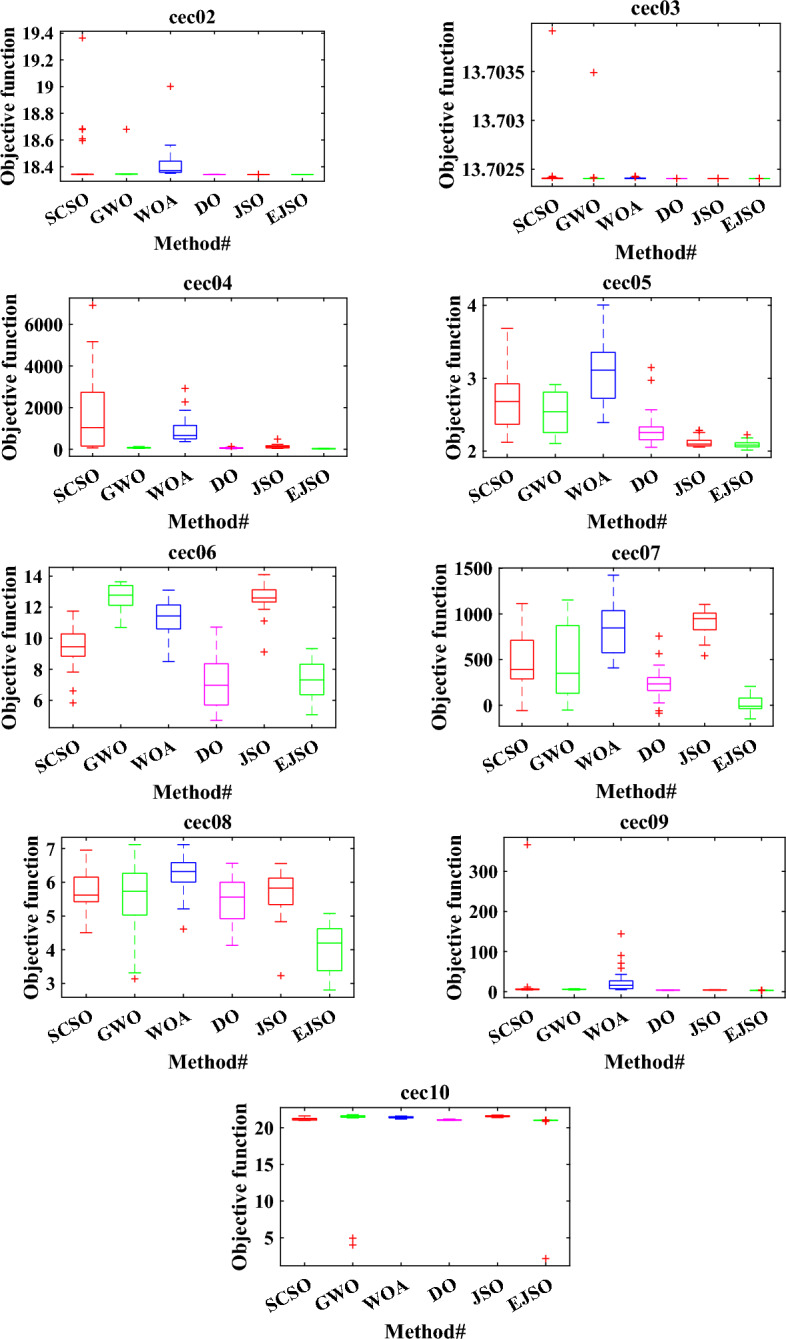
The boxplot of different optimizers.

Boxplot is the best way to display the distribution of the data. The boxplots of the EJSO and the other optimizers are shown in Fig. 8. According to the boxplots, EJSO has the narrowest boxplot compared to the other optimizers for the standard and the CEC 2019 functions compared to GWO, DO, WOA, SCSO, and the standard JSO.

### Application of the EJSO for EM solution

**Figure 9:**
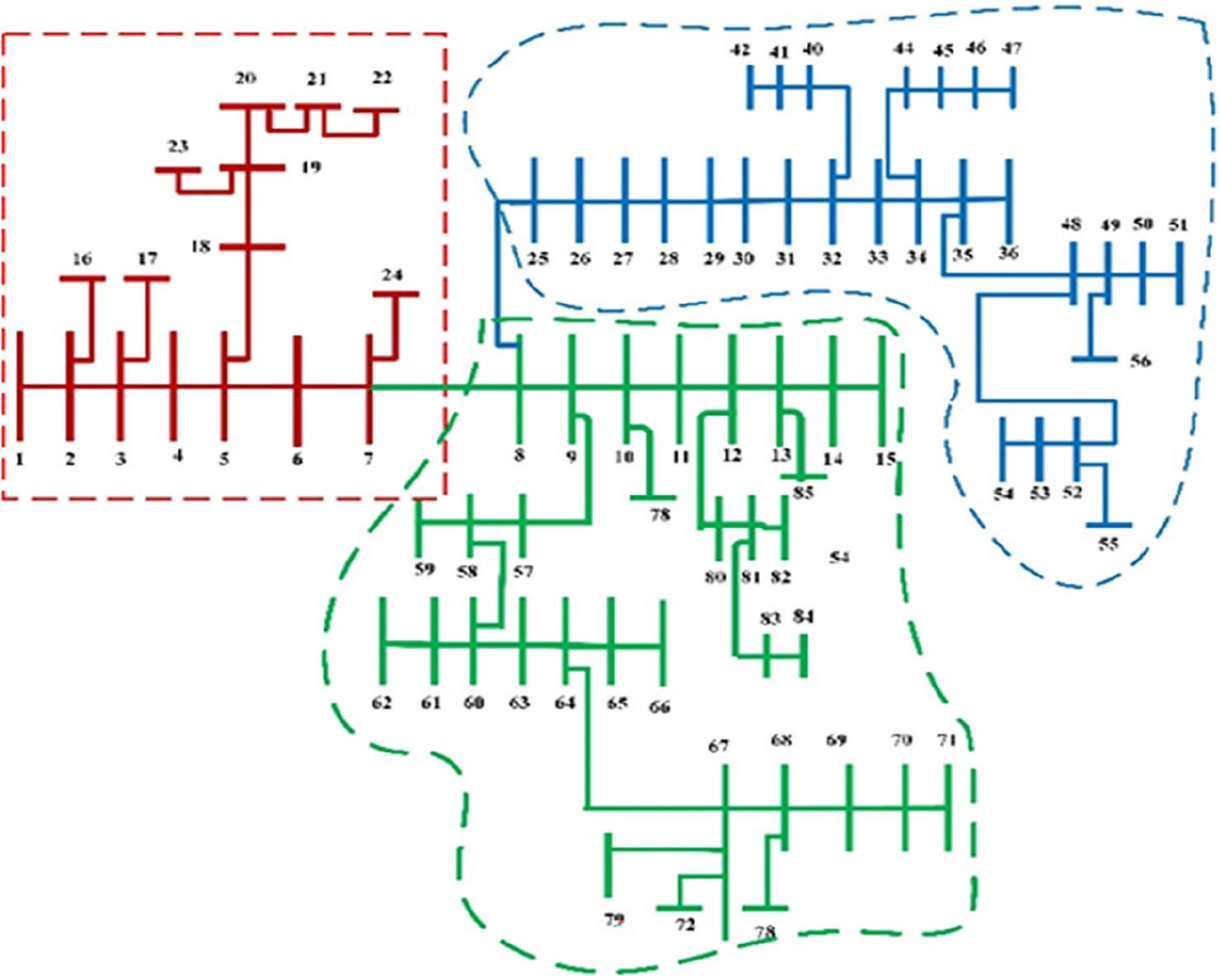
The topology of the IEEE 85-bus MMGs.

**Figure 10:**
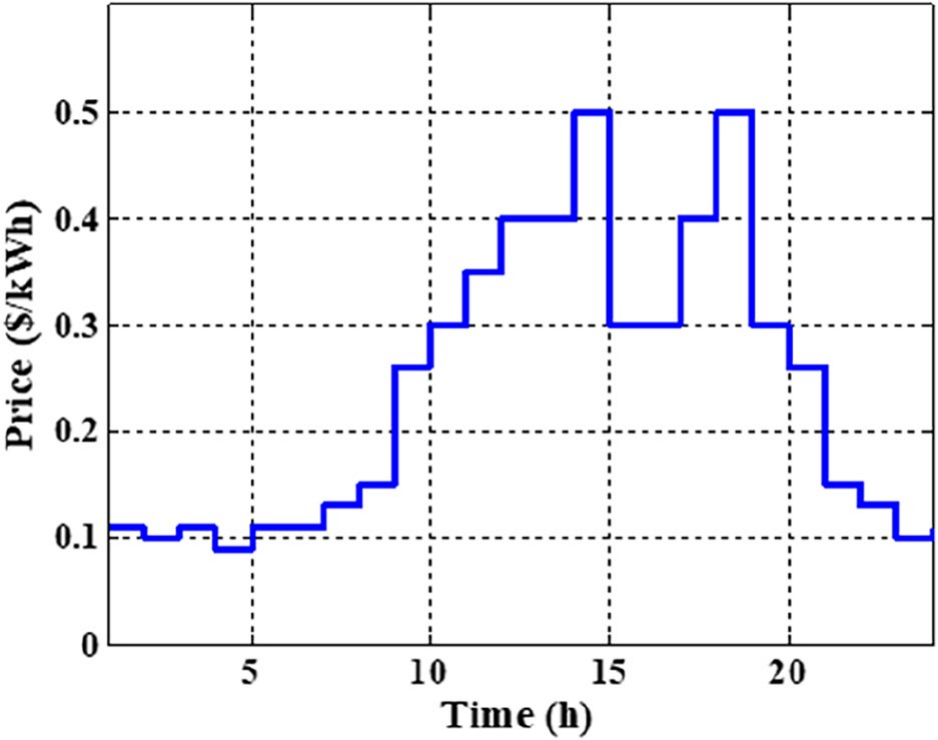
The market energy price.

**Figure 11:**
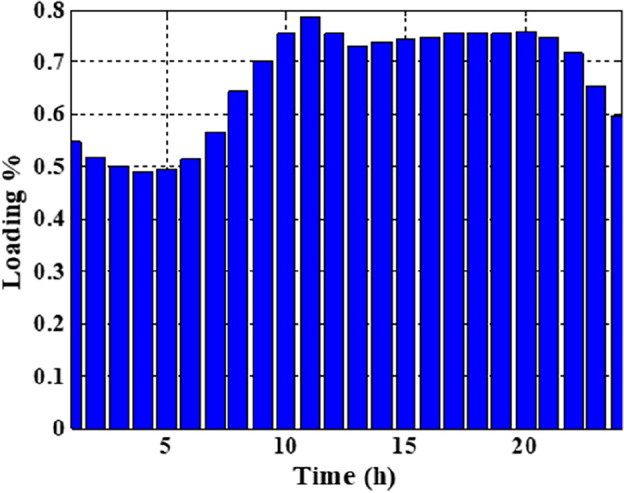
The expected load profile.

**Figure 12:**
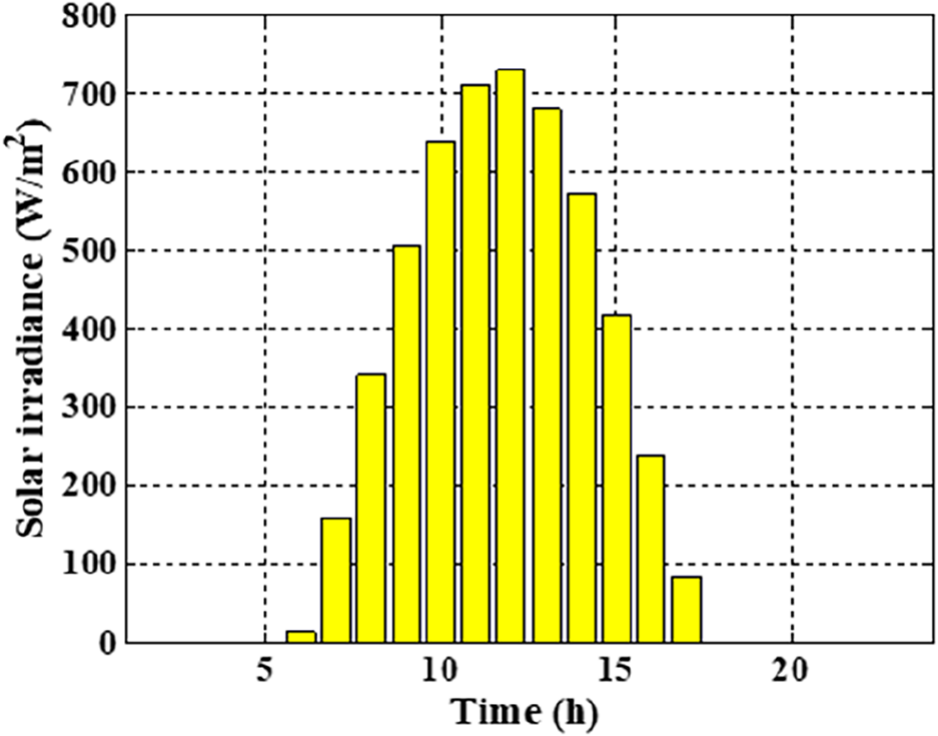
The expected irradiance.

**Figure 13:**
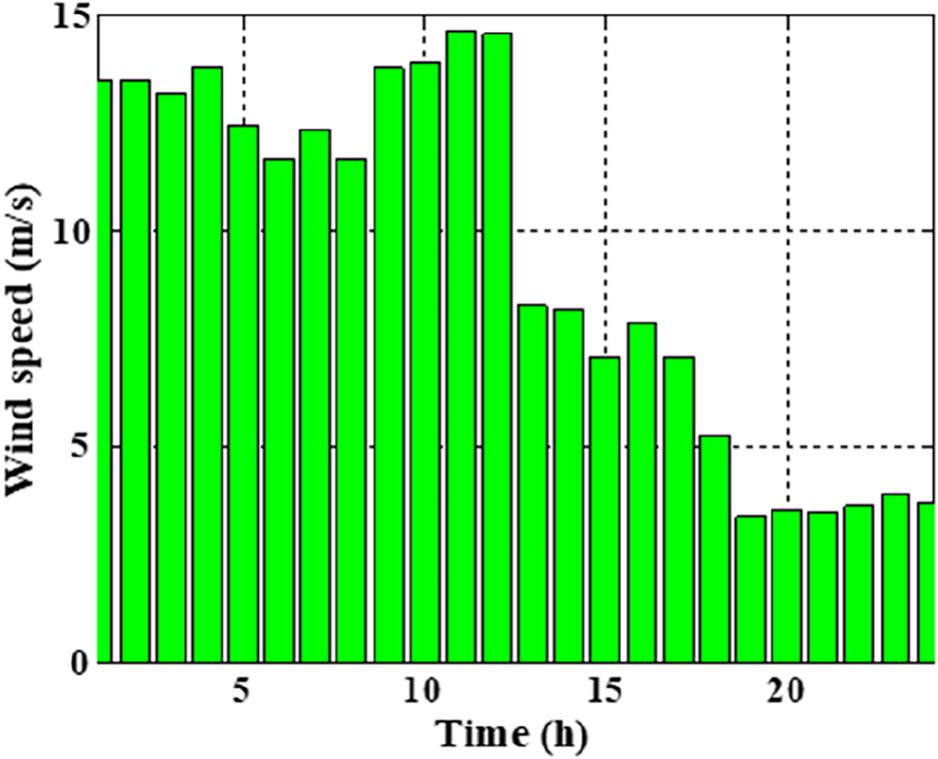
The expected wind speeds.

In this section, the suggested algorithm EJSO has been modified and applied in IEEE 85–bus which is divided into three micro-grids. The description of the IEEE 85-bus is listed in Table 8. The topology of IEEE 85-bus MMGs is depicted in Fig. 9 and lines and bus data are provided in^60^. The studied distribution network has been divided into three microgrids and due to this division, every microgrid contains its RERs (PV, WT, and Biomass) where each network has one PV unit, one WT, and one biomass unit. The captured results by EJSO have been compared with the obtained results by the conventional JSO. For a fair comparison, the populations and the maximum iterations number have been adjusted to be 25 and 80, respectively. The purchasing energy price of the market is explained in Fig. 10 while the day ahead of the loading demand is illustrated in Fig. 11^65^. Figures 12 and 13 show the expected irradiance and wind speed respectively^66^. Three hybrid RERs are incorporated optimally in which each hybrid system consists of a PV plant, a WT, and a biomass generation unit. The system constraints as well as the costs of the PV, WT, and biomass units are listed in Table 9.

**Table 8:** Descriptions of the IEEE 85-bus MMGs.

Parameters	Value
Number of the buses	85
Number of the branches	86
Minimum voltage @ bus	0.87134@ 54
Minimum voltage @ bus	0.99568@2
The real demand	2570 (kW)
The reactive demand	2622 (kVAR)
The real power loss	315 (kW)
The reactive power loss	198.528 (kVAR)
$VD$	119.7076 $(p.u)$
$VSI$	1.5844e+03 $(p.u)$

**Table 9:** The cost parameters and the limitations.

Parameter	Value
PV cost^67^	
$${U}_{PV}$$	770 $ /kW
$${U}_{bio}^{O\&M}$$	0.01 $ /kWh
$${\beta }_{PV}$$	10%
$${NP}_{PV}$$	20
WT cost^68^	
$${U}_{WT}$$	1400 $ /kW
$${U}_{bio}^{O\&M}$$	0.01 $ /kWh
$${\beta }_{WT}$$	10%
$${NP}_{WT}$$	20
Biomass cost^69^	
$${U}_{bio}$$	976 $ /kW
$${U}_{bio}^{O\&M}$$	0.046 $ /kWh
$${\beta }_{Bio}$$	10%
$${NP}_{Bio}$$	20
$${K}_{Loss}$$ ^70^	0.06 $/kWh
The system constraints	
The voltage	$$0.9 p.u\le V\le 1.1 p.u$$
Size of PV units	$$0\le { P}_{PV\_r}\le {P}_{Load}$$
Size of WTs	0 ≤ $${P}_{win{d}_{\_r}}$$≤$${P}_{Load}$$
Size of biomass unit	$$0\le {P}_{bio\_r}\le 200 kW$$
P.F. of the WT	$$0.7\le P.F\le 1$$
P.F. of the Biomass	$$0.7\le P.F\le 1$$

The aim of the EM is total annual cost reduction and the system’s performance improvement. Table 10 lists the numerical results that have been obtained at the base case and with the inclusion of the hybrid PVs, biomass, and WTs using the JSO and the EJSO. The numerical results have been depicted in Table 10 which have been obtained by the JSO and the EJSO for the EM solution with or without RERs. In the base case, the cost, the VDs, and the VSI are 4.1642E+06 USD, 119.7076 p.u. and 1.5844E+03 p.u. respectively while the annual purchased energy and the annual energy losses are 6.4879E+06 kWh and 6.5982e+05 kWh.

**Table 10:** The results of the energy management solution for the MMGs.

	Without RERs	JSO	EJSO
The energy losses (kWh)	1.1671E+06	6.5982E+05	5.2420E+05
The energy loss cost ($)	7.0027E+04	3.9589E+04	3.1452E+04
The purchased energy (kWh)	1.6164E+07	6.4879E+06	6.1429E+06
The cost of the purchased energy ($)	4.0941E+06	1.7013E+06	1.6239E+06
The optimal rating of the PVs (kW)	–	162 226 173	157 151 209
The optimum rating of WTs (kW) and P.F	–	500/0.7635 450/0.8457 450/0.7273	450/0.8710 500/0.7779 500/0.7094
The optimum size (kW)/P.F of the biomass	–	2.5573E+03/0.8480 2.7357E+03/0.7693 3.1334E+03/0.8607	3.6448E+03/0.7676 2.3768E+03/0.7718 2.7403E+03/0.7018
Optimal site of the first hybrid system	–	4	7
Optimal location of the second hybrid system	–	49	55
Optimal location of the third hybrid system	–	69	68
Cost of DGs			
Cost of the first hybrid RESs ($)	–	2.1221E+05	2.0187E+05
Cost of the second hybrid RESs ($)	–	2.0930E+05	2.0798E+05
Cost of the third hybrid RESs ($)	–	2.1021E+05	2.3559E+05
Total cost ($)	4.1642E+06	2.3726E+06	2.3008E+06
$$\sum \text{VD}(\text{p}.\text{u})$$	119.7076	80.9545	70.8672
$$\sum \text{VSI }(\text{p}.\text{u})$$	1.5844E+03	1.7165E+03	1.7517E+03

**Figure 14:**
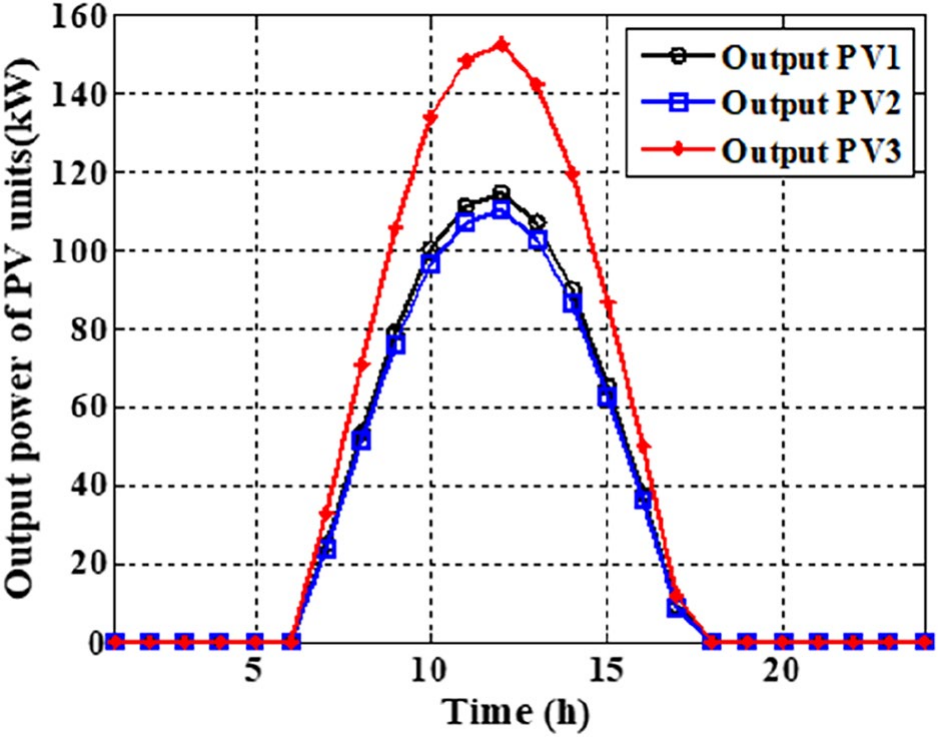
The generated powers from PVs.

**Figure 15:**
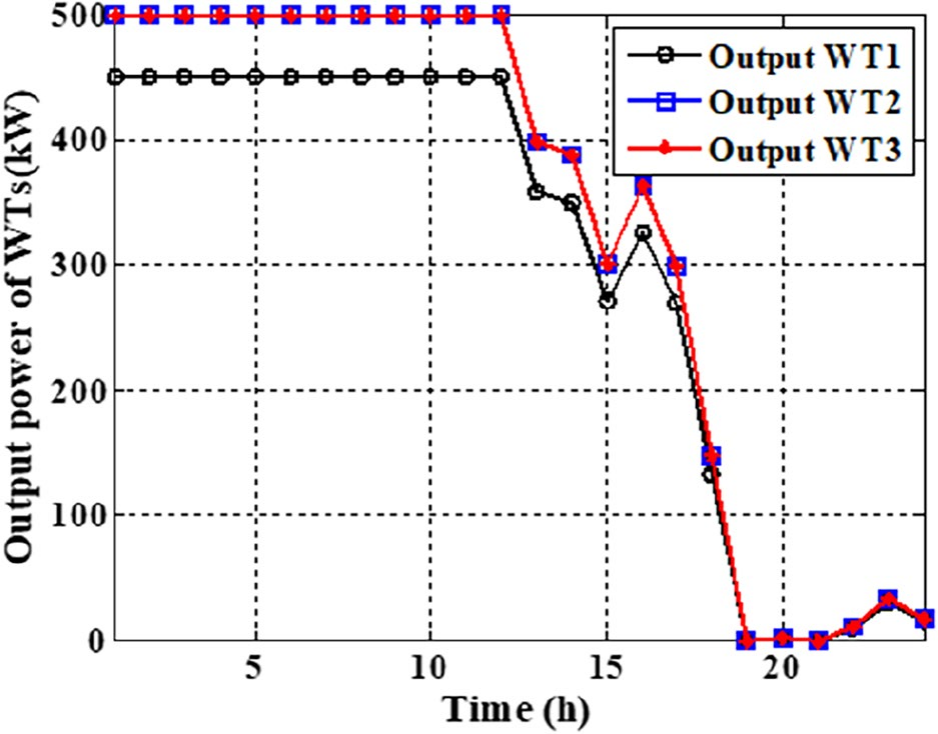
The generated powers from WTs.

**Figure 16:**
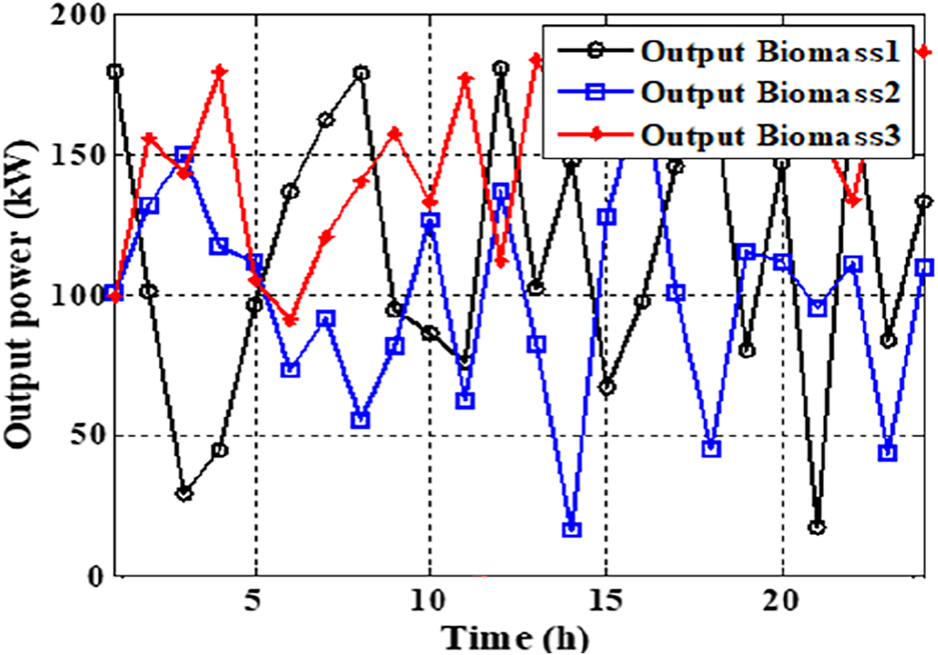
The output power of biomass units.

**Figure 17:**
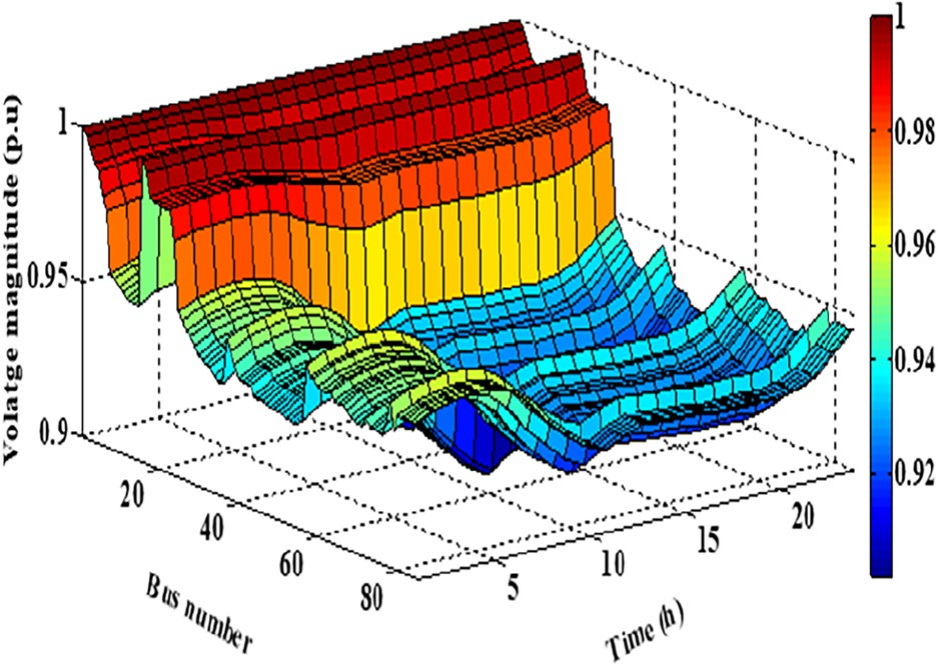
The voltage profile without RERs.

**Figure 18:**
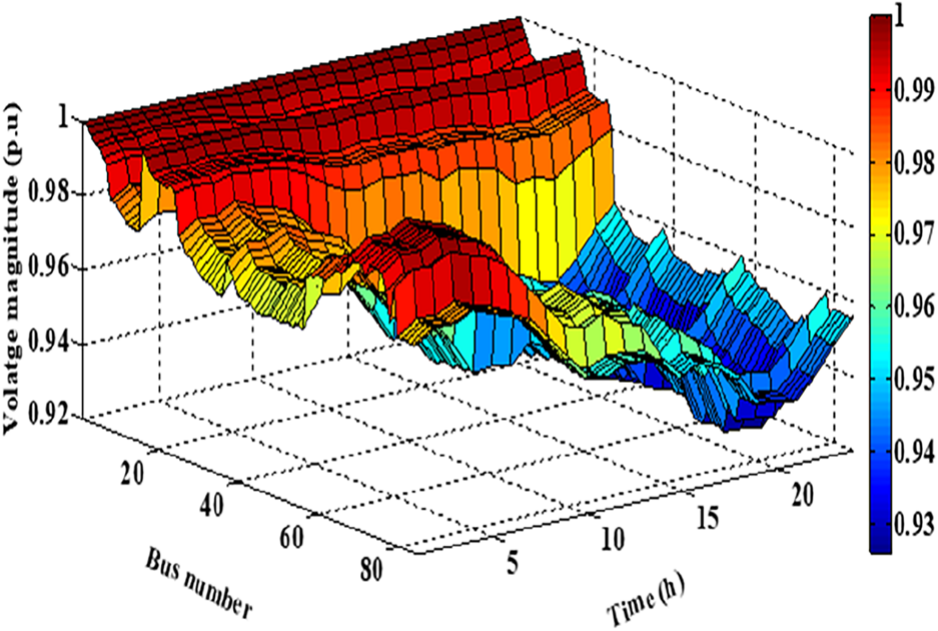
The voltage profile with RERs.

**Figure 19:**
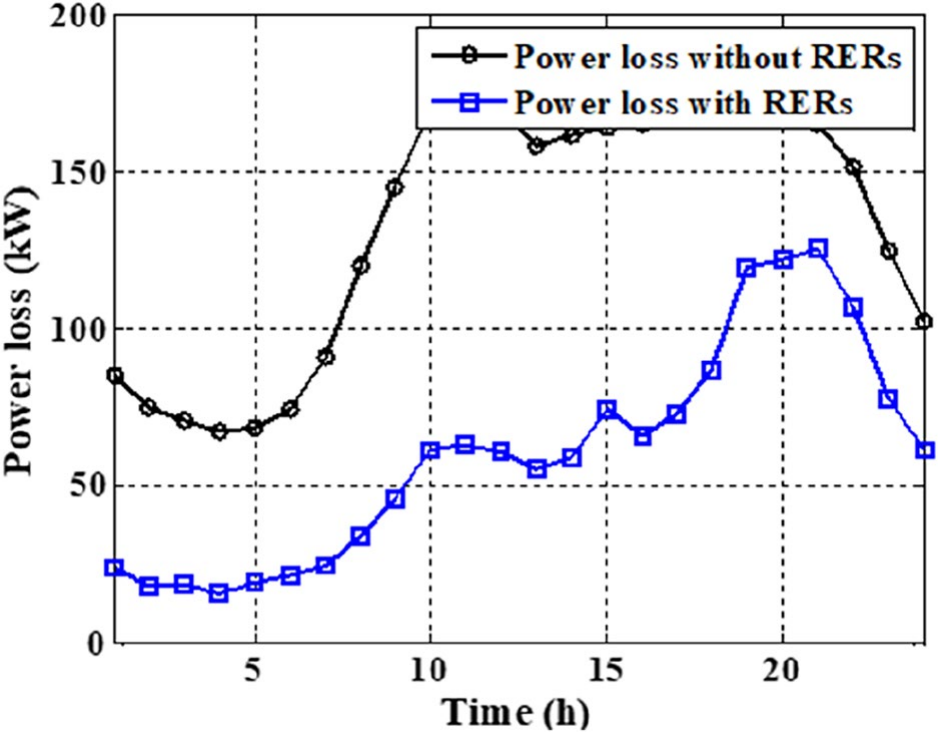
The active losses of the system.

As per the results in Table 10, the total costs have been reduced to 2.3726E+06 p.u. and 2.3008E+0 using the JSO and the EJSO, respectively. Likewise, the summation of VDs has been reduced from 119.7076 p.u. to 70.8672 p.u. and the voltage stability has been enhanced from1.5844E+03 p.u. to 1.7517E+03 p.u. The sites of the three hybrid generation systems that were allocated by JSO are at buses 4, 49, and 69 while the assigned placements by the EJSO are at buses 7, 55, and 68. The optimal ratings of the PV units of the 1^st^, the 2^nd^, and the 3^rd^ hybrid systems that have been determined by the EJSO are 157 kW, 151 kW, and 209 kW, respectively. Likewise, the rating of the WTs in the MMGs are 450 kW, 500 kW, and 500 kW, respectively while the rating of the biomass generation systems are 3.6448E+03 kW, 2.3768E+03 kW, and 2.7403E+03 kW, respectively. The output power of PV units has been illustrated in Fig. 14 according to the solar irradiance as well as Fig. 15 illustrates the output power of the WT units which varied due to the wind speed variation. Figure 16 displays the optimal powers of the biomass system. Figure 17, and Fig. 18 show the voltage profile of the MMG without and with RERs, respectively. From Figs. 17 and 18, it is evident that the voltage profile has been enhanced in the presence of RERs in the proposed hybrid system. As per Fig. 19, the losses were significantly decreased with the installation of the RERs into the system.

## Conclusions

The key findings of this paper can be summarized as follows: firstly, a novel enhanced Jellyfish Search Optimizer (EJSO) was proposed, incorporating Feedback Disturbance Based (FDB) and Weighted Fitness Mechanism (WFM) to address the stagnation issues present in the conventional Jellyfish Search Optimizer (JSO). This enhancement aims to improve the optimization performance and convergence speed of the algorithm.

Secondly, the proposed EJSO was employed for the energy management (EM) of multi-microgrids within an 85-bus system. The optimization process took into consideration various critical factors, including the total cost, voltage profile, and overall stability of the system. The optimal allocation strategy within each microgrid included a hybrid system comprising photovoltaic (PV) systems, wind turbines (WT), and biomass units, which together enhance the efficiency and sustainability of the microgrids.

Moreover, the performance of the proposed EJSO was benchmarked against several other optimization algorithms, including the Sine Cosine Search Optimizer (SCSO), the conventional Jellyfish Search Optimizer (JSO), Differential Evolution (DO), and the Whale Optimization Algorithm (WOA), using both standard test functions and the CEC-2019 benchmark suite. The comparative analysis demonstrated the superiority of EJSO in terms of solution quality and robustness.

In practical terms, the application of the proposed EJSO for energy management with the optimal integration of hybrid Renewable Energy Sources (RESs) significantly reduced the total cost from 4.1642E+06 USD to 2.3008E+06 USD compared to the base case. Additionally, the voltage deviation (VD) was lowered from 119.7076 p.u. to 70.8672 p.u., and the voltage stability index was improved from 1.5844E+03 p.u. to 1.7517E+03 p.u. These results indicate substantial improvements in both economic and technical performance of the microgrid system.

Looking forward, future work associated with this research includes expanding the scope of energy management solutions by integrating electric vehicle (EV) stations into distribution systems. Furthermore, it suggests the optimal incorporation of various types of energy storage systems, such as compressed air energy storage (CAES) and superconducting magnetic energy storage (SMES), to further enhance the flexibility and reliability of the energy management systems.

## Data Availability

The datasets generated during and/or analyzed during the current study are available from the corresponding author on reasonable request.

## References

[1] Ton DT, Smith MA (2012). The US department of energy's microgrid initiative. Electr. J..

[2] Cagnano A, De Tuglie E, Mancarella P (2020). Microgrids: Overview and guidelines for practical implementations and operation. Appl. Energy.

[3] Shahgholian G, Azimi Z (2016). Analysis and design of a DSTATCOM based on sliding mode control strategy for improvement of voltage sag in distribution systems. Electronics.

[4] Natesan C, Ajithan SK, Chozhavendhan S, Devendiran A (2015). Power management strategies in microgrid: A survey. Int. J. Renew. Energy Res..

[5] Bhuyan SK, Hota PK, Panda B (2018). Power quality analysis of a grid-connected solar/wind/hydrogen energy hybrid generation system. Int. J. Power Electron. Drive Syst..

[6] Leonori S, Paschero M, Mascioli FMF, Rizzi A (2020). Optimization strategies for microgrid energy management systems by genetic algorithms. Appl. Soft Comput..

[7] Mokhtara C, Negrou B, Bouferrouk A, Yao Y, Settou N, Ramadan M (2020). Integrated supply–demand energy management for optimal design of off-grid hybrid renewable energy systems for residential electrification in arid climates. Energy Convers. Manag..

[8] Yu, M., Wang, Y. & Li, Y. Energy management of wind turbine-based DC microgrid utilizing modified differential evolution algorithm (2015).

[9] Hachemi AT, Sadaoui F, Saim A, Ebeed M, Abbou HE, Arif S (2023). Optimal operation of distribution networks considering renewable energy sources integration and demand side response. Sustainability.

[10] Hachemi AT (2023). Modified reptile search algorithm for optimal integration of renewable energy sources in distribution networks. Energy Sci. Eng..

[11] Ebeed, M. *et al.* Optimal energy planning of multi-microgrids at stochastic nature of load demand and renewable energy resources using a modified Capuchin Search Algorithm. *Neural Comput. Appl*. 1–26 (2023).

[12] Khunkitti S, Siritaratiwat A, Premrudeepreechacharn S (2022). A many-objective marine predators algorithm for solving many-objective optimal power flow problem. Appl. Sci..

[13] Ezugwu AE, Agushaka JO, Abualigah L, Mirjalili S, Gandomi AH (2022). Prairie dog optimization algorithm. Neural Comput. Appl..

[14] Zhao W, Wang L, Mirjalili S (2022). Artificial hummingbird algorithm: A new bio-inspired optimizer with its engineering applications. Comput. Methods Appl. Mech. Eng..

[15] Fathy A, Abdelaziz AY (2018). Single and multi-objective operation management of micro-grid using krill herd optimization and ant lion optimizer algorithms. Int. J. Energy Environ. Eng..

[16] Ramadan A, Ebeed M, Kamel S, Ahmed EM, Tostado-Véliz M (2023). Optimal allocation of renewable DGs using artificial hummingbird algorithm under uncertainty conditions. Ain Shams Eng. J..

[17] Abid MS, Apon HJ, Morshed KA, Ahmed A (2022). Optimal planning of multiple renewable energy-integrated distribution system with uncertainties using artificial hummingbird algorithm. IEEE Access.

[18] Abid MS, Ahshan R, Al Abri R, Al-Badi A, Albadi M (2023). Multi-objective optimal planning of virtual synchronous generators in microgrids with integrated renewable energy sources. IEEE Access.

[19] Ahmed D, Ebeed M, Ali A, Alghamdi AS, Kamel S (2021). Multi-objective energy management of a micro-grid considering stochastic nature of load and renewable energy resources. Electronics.

[20] Zhou B (2021). Multi-microgrid energy management systems: Architecture, communication, and scheduling strategies. J. Modern Power Syst. Clean Energy.

[21] Ma G, Li J, Zhang X-P (2022). A review on optimal energy management of multimicrogrid system considering uncertainties. IEEE Access.

[22] Xiong L (2022). A two-level energy management strategy for multi-microgrid systems with interval prediction and reinforcement learning. IEEE Trans. Circuits Syst. I Regul. Pap..

[23] Wenzhi S, Zhang H, Tseng M-L, Weipeng Z, Xinyang L (2022). Hierarchical energy optimization management of active distribution network with multi-microgrid system. J. Ind. Prod. Eng..

[24] Rahnama A, Shayeghi H, Dejamkhooy A, Bizon N (2022). A cost-technical profit-sharing approach for optimal energy management of a multi-microgrid distribution system. Energy.

[25] Ahmadi SE, Rezaei N (2020). A new isolated renewable based multi microgrid optimal energy management system considering uncertainty and demand response. Int. J. Electr. Power Energy Syst..

[26] Wang Z, Chen B, Wang J, Begovic MM, Chen C (2014). Coordinated energy management of networked microgrids in distribution systems. IEEE Trans. Smart Grid.

[27] Abd El-Sattar H, Sultan HM, Kamel S, Khurshaid T, Rahmann C (2021). Optimal design of stand-alone hybrid PV/wind/biomass/battery energy storage system in Abu-Monqar, Egypt. J. Energy Storage.

[28] Behera BK, Varma A (2016). Microbial Resources for Sustainable Energy.

[29] Samy M, Elkhouly HI, Barakat S (2021). Multi-objective optimization of hybrid renewable energy system based on biomass and fuel cells. Int. J. Energy Res..

[30] Al-Ghussain L, Ahmad AD, Abubaker AM, Mohamed MA (2021). An integrated photovoltaic/wind/biomass and hybrid energy storage systems towards 100% renewable energy microgrids in university campuses. Sustain. Energy Technol. Assess..

[31] Naraharisetti PK, Karimi I, Anand A, Lee D-Y (2011). A linear diversity constraint–application to scheduling in microgrids. Energy.

[32] Wang C, Liu Y, Li X, Guo L, Qiao L, Lu H (2016). Energy management system for stand-alone diesel-wind-biomass microgrid with energy storage system. Energy.

[33] Rashid, A., Hasan, N., Parvez, K. T. & Maruf, M. N. I. Study and analysis of a small scale micro-grid using renewable energy resources. In *2015 International Conference on Electrical Engineering and Information Communication Technology (ICEEICT)*, 1–4 (IEEE, 2015).

[34] Abo-Elyousr FK, Elnozahy A (2018). Bi-objective economic feasibility of hybrid micro-grid systems with multiple fuel options for islanded areas in Egypt. Renew. Energy.

[35] Balamurugan P, Ashok S, Jose T (2009). Optimal operation of biomass/wind/PV hybrid energy system for rural areas. Int. J. Green Energy.

[36] Kahraman HT, Aras S, Gedikli E (2020). Fitness-distance balance (FDB): A new selection method for meta-heuristic search algorithms. Knowl.-Based Syst..

[37] Bakır H (2024). Dynamic fitness-distance balance-based artificial rabbits optimization algorithm to solve optimal power flow problem. Expert Syst. Appl..

[38] Bakir H, Guvenc U, Kahraman HT, Duman S (2022). Improved Lévy flight distribution algorithm with FDB-based guiding mechanism for AVR system optimal design. Comput. Ind. Eng..

[39] Bakır H, Duman S, Guvenc U, Kahraman HT (2023). Improved adaptive gaining-sharing knowledge algorithm with FDB-based guiding mechanism for optimization of optimal reactive power flow problem. Electr. Eng..

[40] Ebeed M (2024). A Modified Artificial Hummingbird Algorithm for solving optimal power flow problem in power systems. Energy Rep..

[41] Layeb, A. Differential evolution algorithms with novel mutations, adaptive parameters, and Weibull flight operator. *Soft Comput.* 1–53 (2024).

[42] Ebeed M, Alhejji A, Kamel S, Jurado F (2020). Solving the optimal reactive power dispatch using marine predators algorithm considering the uncertainties in load and wind-solar generation systems. Energies.

[43] Abdel-Fatah, S., Ebeed, M., Kamel, S. & Nasrat, L. Moth swarm algorithm for reactive power dispatch considering stochastic nature of renewable energy generation and load. In *2019 21st International Middle East Power Systems Conference (MEPCON)*, 594–599 (IEEE, 2019).

[44] Ali E, Abd Elazim S, Abdelaziz A (2016). Ant lion optimization algorithm for renewable distributed generations. Energy.

[45] Ebeed M, Mostafa A, Aly MM, Jurado F, Kamel S (2023). Stochastic optimal power flow analysis of power systems with wind/PV/TCSC using a developed Runge Kutta optimizer. Int. J. Electr. Power Energy Syst..

[46] Biswas PP, Suganthan PN, Mallipeddi R, Amaratunga GA (2019). Optimal reactive power dispatch with uncertainties in load demand and renewable energy sources adopting scenario-based approach. Appl. Soft Comput..

[47] Karaki S, Chedid R, Ramadan R (1999). Probabilistic performance assessment of autonomous solar-wind energy conversion systems. IEEE Trans. Energy Convers..

[48] Ebeed M, Shady H, Aleem A (2021). Chapter 1—Overview of Uncertainties in Modern Power Systems: Uncertainty Models and Methods, Uncertainties in Modern Power Systems.

[49] Kayal P, Chanda C (2015). Optimal mix of solar and wind distributed generations considering performance improvement of electrical distribution network. Renew. Energy.

[50] Zio E, Zio E (2013). Monte Carlo Simulation: The Method.

[51] Growe-Kuska, N., Heitsch, H. & Romisch, W. Scenario reduction and scenario tree construction for power management problems. In *2003 IEEE Bologna Power Tech Conference Proceedings*, Vol. 3, 7 (IEEE, 2003).

[52] Chou J-S, Truong D-N (2021). A novel metaheuristic optimizer inspired by behavior of jellyfish in ocean. Appl. Math. Comput..

[53] Weibull W (1951). A statistical distribution function of wide applicability. J. Appl. Mech..

[54] Aras S, Gedikli E, Kahraman HT (2021). A novel stochastic fractal search algorithm with fitness-distance balance for global numerical optimization. Swarm Evol. Comput..

[55] Guvenc U, Duman S, Kahraman HT, Aras S, Katı M (2021). Fitness–Distance Balance based adaptive guided differential evolution algorithm for security-constrained optimal power flow problem incorporating renewable energy sources. Appl. Soft Comput..

[56] Sharifi MR, Akbarifard S, Qaderi K, Madadi MR (2021). Developing MSA algorithm by new fitness-distance-balance selection method to optimize cascade hydropower reservoirs operation. Water Resour. Manag..

[57] Zheng K, Yuan X, Xu Q, Dong L, Yan B, Chen K (2022). Hybrid particle swarm optimizer with fitness-distance balance and individual self-exploitation strategies for numerical optimization problems. Inf. Sci..

[58] Mirjalili S, Mirjalili SM, Lewis A (2014). Grey wolf optimizer. Adv. Eng. Softw..

[59] Mirjalili S, Lewis A (2016). The whale optimization algorithm. Adv. Eng. Softw..

[60] Seyyedabbasi, A. & Kiani, F. Sand Cat swarm optimization: A nature-inspired algorithm to solve global optimization problems. *Eng. Comput*. 1–25 (2022).

[61] Zhao S, Zhang T, Ma S, Chen M (2022). Dandelion optimizer: A nature-inspired metaheuristic algorithm for engineering applications. Eng. Appl. Artif. Intell..

[62] Jamil, M. & Yang, X.-S. A literature survey of benchmark functions for global optimization problems. arXiv preprint arXiv:1308.4008, (2013).

[63] Molga, M. & Smutnicki, C. *Test Functions for Optimization Needs* Vol. 101, 48, (2005).

[64] Ahmadi B, Giraldo JS, Hoogsteen G (2023). Dynamic Hunting Leadership optimization: Algorithm and applications. J. Comput. Sci..

[65] Zhang Y, Ren S, Dong ZY, Xu Y, Meng K, Zheng Y (2017). Optimal placement of battery energy storage in distribution networks considering conservation voltage reduction and stochastic load composition. IET Gener. Transm. Distrib..

[66] Moradi MH, Abedini M, Tousi SR, Hosseinian SM (2015). Optimal siting and sizing of renewable energy sources and charging stations simultaneously based on differential evolution algorithm. Int. J. Electr. Power Energy Syst..

[67] Gampa SR, Das D (2015). Optimum placement and sizing of DGs considering average hourly variations of load. Int. J. Electr. Power Energy Syst..

[68] Augustine, N., Suresh, S., Moghe, P. & Sheikh, K. Economic dispatch for a microgrid considering renewable energy cost functions. In *2012 IEEE PES Innovative Smart Grid Technologies (ISGT)*, 1–7 (IEEE, 2012).

[69] Abo El-Ela AA, Allam SM, Shaheen AM, Nagem NA (2021). Optimal allocation of biomass distributed generation in distribution systems using equilibrium algorithm. Int. Trans. Electr. Energy Syst..

[70] Sultana S, Roy PK (2014). Optimal capacitor placement in radial distribution systems using teaching learning based optimization. Int. J. Electr. Power Energy Syst..

